# Use of bailing capsules (cordyceps sinensis) in the treatment of chronic kidney disease: a meta-analysis and network pharmacology

**DOI:** 10.3389/fphar.2024.1342831

**Published:** 2024-04-05

**Authors:** Yilin Tao, Ruixiang Luo, Yuanbing Xiang, Min Lei, Xuan Peng, Yao Hu

**Affiliations:** ^1^ Department of Medicine Renal Division, Affiliated Hospital & Clinical Medical College of Chengdu University, Chengdu, China; ^2^ The Third Affiliated Hospital of Sun Yat Sen University, Guangzhou, China; ^3^ Department of Medicine Renal Division, West China Hospital, West China School of Medicine, Sichuan University, Chengdu, China

**Keywords:** bailing capsules, cordyceps sinensis, chronic kidney disease, meta-analysis, network pharmacology

## Abstract

The Bailing Capsule is a commonly used traditional Chinese medicine for the treatment of chronic kidney disease (CKD). However, its therapeutic effects and pharmacological mechanisms have not been fully explored. In this study, we integrated meta-analysis and network pharmacology to provide scientific evidence for the efficacy and pharmacological mechanism of Bailing Capsule in treating CKD. We conducted searches for randomized controlled studies matching the topic in PubMed, the Cochrane Library, Embase, Web of Science, and the Wanfang Database, and screened them according to predefined inclusion and exclusion criteria. Dates from the included studies were extracted for meta-analysis, including renal function indicators, such as 24-h urinary protein (24UP), blood urea nitrogen (BUN), and serum creatinine (Scr), as well as inflammatory indicators like high-sensitivity C-reactive protein (hs-CRP), interleukin-6 (IL-6), and tumor necrosis factor-alpha (TNF-α). Network pharmacology was employed to extract biological information, including active drug ingredients and potential targets of the drugs and diseases, for network construction and gene enrichment. Our findings indicated that 24UP, BUN, and Scr in the treatment group containing Bailing Capsule were lower than those in the control group. In terms of inflammatory indicators, hs-CRP, IL-6, and TNF-α, the treatment group containing Bailing Capsule also exhibited lower levels than the control group. Based on network pharmacology analysis, we identified 190 common targets of Bailing Capsule and CKD. Gene Ontology (GO) and Kyoto Encyclopedia of Genes and Genomes (KEGG) analyses suggested that the pharmacological mechanism of Bailing Capsule might be related to immune response, inflammatory response, vascular endothelial damage, cell proliferation, and fibrosis. This demonstrates that Bailing Capsule can exert therapeutic effects through multiple targets and pathways, providing a theoretical basis for its use.

## 1 Introduction

Chronic kidney disease (CKD) poses a significant threat to human health. Currently, the number of CKD patients is increasing due to rising risk factors such as diabetes, hypertension, and obesity. In 2017, an estimated 843.6 million people worldwide were affected by CKD, making it one of the most prevalent diseases globally (GBD, 2017 Diet Collaborators, 2019). And CKD patients in China accounted for nearly one-fifth of the world’s, and became the country with the most CKD patients. Between 1990 and 2019, the prevalence and mortality rate of CKD increased significantly (Li et al., 2023). In 2019, there were 150.5 million cases of (10.6%) and 196 726 deaths from (13.8 per 100 000 general population) CKD in China (Li et al., 2023). And the CKD prevalence and mortality are projected to rise to 11.7% and 17.1 per 100 000, respectively, by 2029 (Li et al., 2023). A horizontal section study showed that among Chinese adult CKD patients, 73.3%, 25.0% and 1.8% were at stage 1 to 2, 3, and 4 to 5, respectively, and the awareness of CKD was 10.0% (Wang et al., 2023). It can be seen that most of the patients with CKD are in the early stages. Therefore, the prevention and control of early CKD and delaying the development of the disease are very important.

Currently, the primary intervention measures for CKD stages 1 and 2 involve managing hypertension and its associated complications, utilizing ACE inhibitors (ACEI) or angiotensin II receptor blockers (ARB) to inhibit the renin-angiotensin system (RAS) and reduce the risk of cardiovascular disease. In CKD stage 3, comprehensive evaluation and treatment of other complications resulting from reduced glomerular filtration rate (GFR) are crucial. This may include addressing issues like anemia, disturbances in calcium and phosphorus metabolism, and renal osteodystrophy, among others. When CKD progresses to stage 4, preparations for renal replacement therapy should be initiated. Renal replacement therapy (RRT) can be employed when uremic symptoms manifest (Levey et al., 2003). One of the biggest improvements in CKD treatment in the past 10 years is to find that SGLT-2 inhibitors have a strong protective effect on the heart and kidneys of patients with or without diabetes (Usman et al., 2023). The application of SGLT-2 can bring clinical benefits to early CKD patients (The et al., 2023), and even CKD patients who have severely damaged renal function may also have certain potential benefits (Heerspink et al., 2023a). GLP-1 receptor agonists have also demonstrated efficacy in improving renal outcomes among patients with type 2 diabetes, albeit within trials designed primarily for cardiac endpoints (Sattar et al., 2021). Finerenone, a non-steroidal selective MRA, was also recently approved to treat CKD for potentially greater anti-inflammatory and antifibrotic effects (Barrera-Chimal et al., 2022; Epstein et al., 2022; Lo et al., 2023). Sparsentan, a dual endothelin and angiotensin II receptor antagonist, is also being investigated as a treatment for FSGS and IgA nephropathy (Heerspink et al., 2019; Heerspink et al., 2023b). However, due to side effects, the use of drugs has been limited, such as hypertopolymia, and aggravated renal function damage.

The treatment about TCM has always been controversial. In China and other Asian countries, TCM is widely used in CKD patients to delay kidney failure. However, in Western countries, because the components and efficacy of TCM are not completely clear, it is not recommended in the guidelines. With the advancement of TCM, numerous studies at the molecular level have also provided substantial evidence for the efficacy and mechanisms of TCM (Lu et al., 2019; Ma et al., 2023). For example, in the recently published RCT study of Shenyankangfu Tablet in the treatment of primary glomerulonephritis, Shenyankangfu Tablet decreased the proteinuria (Wu et al., 2021). And Huangkui Capsule (HKC) may reduce podocyte damage to ameliorate proteinuria via JAK2/STAT3 and PI3K/Akt pathway (Zhao et al., 2023).

The Bailing Capsule is TCM which is refined by low-temperature fermentation of cordyceps strains. In clinical treatment, Bailing capsule as an adjuvant therapy, can be applied to chronic renal insufficiency caused by various reasons, such as glomerulonephritis, diabetic nephropathy, nephrotic syndrome, lupus nephritis. Meta-analyses have shown that the Bailing capsule, combined with Western medicine, has better efficacy than Western medicine alone by reducing urine protein and protecting kidney function (Li et al., 2020; Sheng et al., 2020; Zhao et al., 2022). And it shown that cordyceps sinensis have effects of anti-hyperglycemic, anti-inflammatory, immunomodulatory, antioxidant, anti-fibrotic activities (Zhang et al., 2014; Tan et al., 2022). Therefore, Bailing capsule mainly plays a therapeutic role through cordyceps sinensis. In this study, we employed meta-analysis and network pharmacology methodologies to construct an objective and comprehensive assessment of Bailing Capsule’s therapeutic potential. This systematic review and elucidation of its mechanisms offer valuable insights for the clinical application of Bailing Capsules.

## 2 Materials and methods

### 2.1 Search strategy

We systematically searched relevant databases, including PubMed, the Cochrane Library, Embase, Web of Science, and the Wanfang Database, from their respective inception dates up to December 2023. This search employed a combination of subject terms and keywords. In English, the search terms encompassed “Cordyceps (Mesh)," “Bailing Capsule,” “Corbrin Capsule,” “traditional Chinese medicine,” and “Renal Insufficiency, Chronic (Mesh)" In Chinese, the search terms included “chronic kidney disease,” “chronic renal insufficiency,” “chronic renal failure,” “chronic glomerulonephritis,” “uremia,” “Bailing Capsule,” “Bailing,” “Cordyceps sinensis,” and “Chinese patent medicine.” Keywords within the same category of search terms were combined using “or,” and the connection between drugs and diseases was established using “and”.

### 2.2 Inclusion and exclusion criteria

Inclusion criteria: 1) Patient Population: Patients diagnosed with CKD which is defined as the presence of renal damage persisting for at least 3 months, with or without a decline in glomerular filtration rate (GFR), and a GFR of less than 60 mL/min/1.73 m^2^ for a duration of at least 3 months. 2) Treatment Interventions: The control group received contemporary Western medicine, typically ARB or ACEI drugs. The experimental group received Bailing Capsules as a standalone treatment or in combination with the drugs prescribed to the control group, with the experimental group being administered Bailing Capsules in addition to the control group’s regimen. The duration of treatment was not limited. 3) Study Outcomes: The outcomes indicators included any of the following: renal function indicators 24-h urine protein, BUN, and Scr, and inflammatory markers like high-sensitivity hs-CRP, IL-6, and TNF-α. 4) Study Design: Randomized controlled trials. 5) No restrictions were placed on sample size or follow-up duration.

Exclusion Criteria: 1) The study population must meet the diagnostic criteria for CKD; unclear or undocumented diagnoses, as mentioned in the article but not specified in the text, are excluded. 2) CKD patients applied alternative treatments such as dialysis or kidney transplantation are excluded. 3) Non-clinical randomized controlled trials, such as case reports, animal experiments, reviews, and duplicate published studies, are excluded. 4) Primary outcome measures that do not align with the outcome measures of our investigation are excluded.

### 2.3 Literature screening and data extraction

Two authors independently conducted a thorough assessment of the retrieved literature, meticulously following the predetermined inclusion and exclusion criteria, in order to identify eligible studies and extract crucial data pertaining to patients’ baseline characteristics, dosage information, and outcome indicators. The extracted data from the literature encompassed the author’s name, year of publication, essential patient demographics (age and gender), treatment approaches employed in both the control and experimental groups, treatment duration, and the sample size. Outcome measures encompassed key parameters such as BUN, 24hUP, Scr, as well as the inflammatory markers TNF-α, IL-6, and hs-CRP.

### 2.4 Methodological quality assessment

The quality of the literature was assessed using the Cochrane Collaboration Assessment Tool, which evaluated the risk of bias across six domains: random sequence generation (selection bias), allocation concealment (selection bias), blinding of participants and personnel (performance bias), blinding of outcome assessment (detection bias), incomplete outcome data (attrition bias), selective reporting (reporting bias), and other potential biases. The assessments resulted in categorizations of low risk, unclear risk, or high risk.

### 2.5 Data analysis

Data analysis was performed using Revman 5.3. For continuous variables, the Standardized Mean Difference (SMD) was employed, with the calculation of 95% confidence intervals (CI). Heterogeneity was assessed using I2. When there was no significant heterogeneity in the combined data (*p* ≥ 0.10, I2 ≤ 50%), a fixed-effects model was used for meta-analysis. In cases of significant heterogeneity in the data (*p* ≤ 0.10, I2 ≥ 50%), meta-analysis was conducted using a random-effects model. For studies displaying heterogeneity, a sensitivity meta-analysis was carried out by systematically excluding literature that did not meet the inclusion criteria one by one. Qualitative and quantitative assessments of publication bias were performed using funnel plots, Begg’s rank correlation, and Egger’s regression.

### 2.6 Network pharmacology method of bailing for CKD

The Traditional Chinese Medicine Systems Pharmacology (TCMSP) database is routinely utilized to retrieve information about the ingredients and target proteins of Chinese Materia Medica. In our study, we initially conducted a search for the active constituents and target proteins of Bailing within the TCMSP database. Additionally, we conducted supplementary searches in PubMed, CNKI, and other relevant sources. To identify the gene names associated with these target proteins, we turned to the UniProt database. Using the R project software, we meticulously refined and filtered the gathered gene names, ultimately enabling us to identify the active ingredients of Bailing and their corresponding target genes.

### 2.7 Collation of target genes related to human CKD

Search with the keyword “CKD” (Chronic kidney disease)in Gencards. Subsequently, we meticulously compiled the relevant gene names and employed R software to identify target genes associated with CKD. Utilizing the R project software, we then determined the intersection between the target genes of Bailing Capsules’ active ingredients and those genes related to chronic kidney disease, culminating in the construction of a Venn diagram. The shared targets identified in this analysis were subsequently recognized as the key targets for Bailing Capsules in the treatment of CKD. Moreover, we imported these common target genes into Cytoscape 3.8.2 software to create a component-target-disease relationship network, facilitating a comprehensive understanding of the intricate relationships between the active constituents, targets, and the disease dynamics attributed to Bailing Capsules.

### 2.8 Building protein-protein interaction (PPI) network

The common target genes we obtained were organized into a protein-protein interaction (PPI) network using the String network database, with the criteri “minimum required interaction score: Highest confidence (0.900)” applied to protein-protein interactions. After constructing the PPI network, we systematically removed irrelevant protein nodes, isolated the relevant nodes, saved them as TSV format files, and subsequently imported the data into Cytoscape 3.8.2 software for a thorough analysis of the PPI network’s topology.

### 2.9 GO bioanalysis and KEGG pathway analysis

We utilized the R programming language to perform Gene Ontology (GO) and Kyoto Encyclopedia of Genes and Genomes (KEGG) analyses on the overlapping genes we obtained. We then visually represented the top 20 results using both bar charts and bubble charts. These analyses aimed to uncover the primary molecular biological processes and signaling pathways associated with the key targets.

## 3 Results

### 3.1 Research search and screening

**FIGURE 1:**
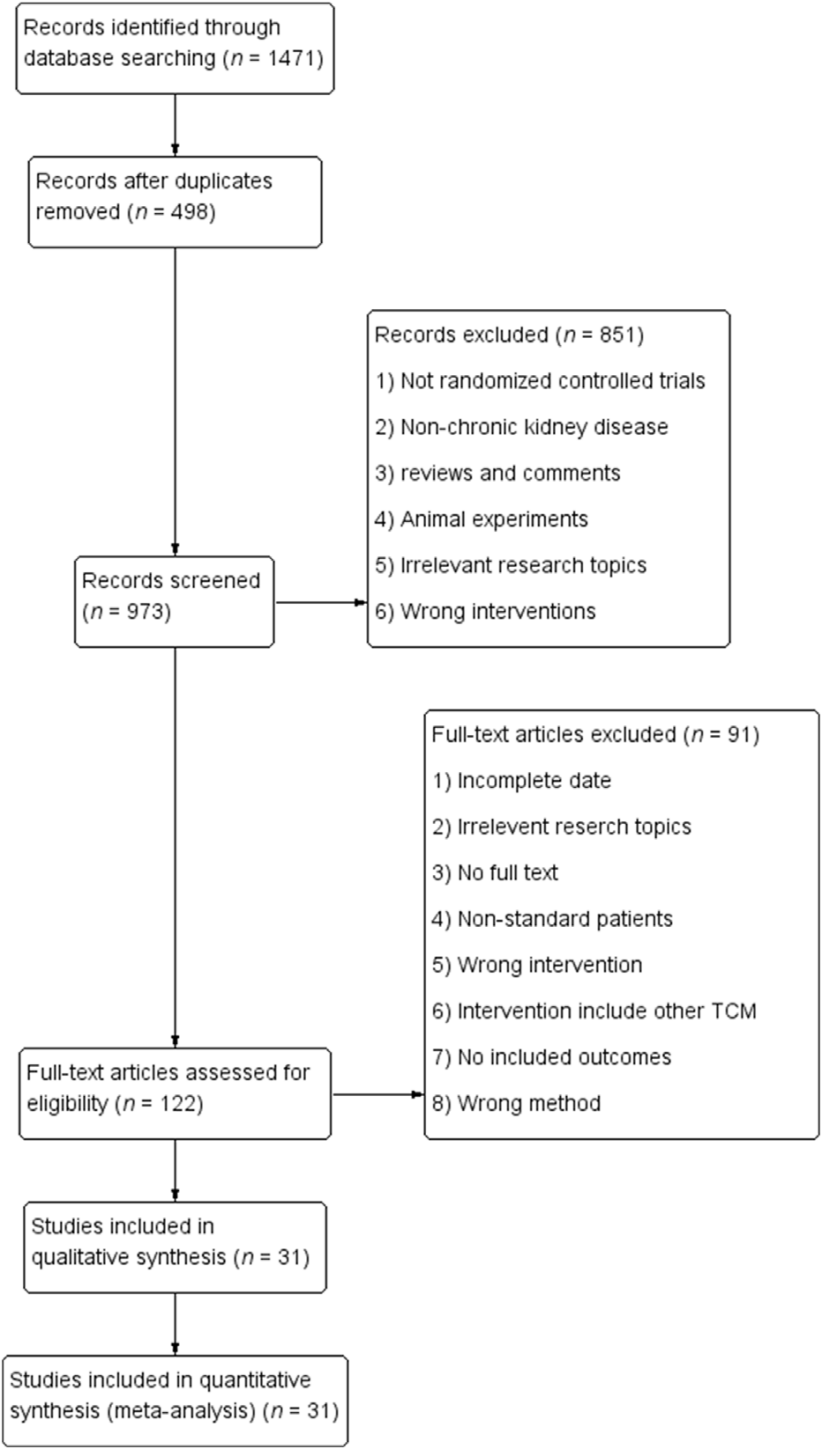
Flow chart of Literature screening.

We initially retrieved a total of 1,471 articles and eliminated 498 duplicates. After conducting a comprehensive evaluation of abstracts and titles, we excluded 58 articles that were not in line with the objectives of the study. Additionally, 375 other articles, including meta-analyses, reviews, meeting abstracts, case reports, and animal experiments, were excluded. The stipulated intervention criteria were not met by an additional 170 articles. After conducting a comprehensive evaluation of the complete texts and strictly adhering to our predetermined inclusion and exclusion criteria, we ultimately excluded 91 articles, resulting in the selection of 31 randomized controlled trial (RCT) articles (Liu, 2006; Yang et al., 2009; Huang et al., 2014; Liu and Liu, 2015; Liu et al., 2017; Zhu et al., 2017; Deng and Wang, 2018; He et al., 2018; Feng et al., 2019; Li et al., 2019; Qiu, 2019; Song, 2019; Tang and Xu, 2019; Yu et al., 2019; Zheng et al., 2019; Liu, 2021a; Zhang, 2021a; Liu, 2021b; Zhang, 2021b; Jia et al., 2021; Ma and Yan, 2021; Sun and Zhao, 2021; Wang, 2021; Chen and Xu, 2022; Guo, 2022; Quan et al., 2022; Shao and Yu, 2022; Tang, 2022; Zhang, 2022; Cui et al., 2023; Hu, 2023). A detailed breakdown of the article screening process and results is provided in Figure 1. Among the 31 included articles, 21 studies examined the coadministration of Bailing Capsules with ARB/ACEI/ARNI drugs, while the remaining 11 studies investigated the combined use of Bailing capsules with conventional medical treatments, such as α-ketoacid tablets, alprostadil, ferulic tablets, cyclophosphamide, or unspecified regimens. More information regarding the included studies is presented in Table 1. And it shown that the chronic glomerulonephritis (CGN) was the most common disease type in the included studies, followed by diabetic nephropathy (DN) and hypertensive nephropathy (HN).

**TABLE 1:** Characteristics of included studies.

	Study	Male	Mean age(y)	Included disease	Interventions	Bailing dose	Treatment Duration(w)	Outcomes
1	Chen and Xu. (2022)	C: 31	C:46.9 ± 10.7	CGN	C: ARB	2.5 g, tid	8	24 hUP; BUN; Scr
		T: 29	T:47.4 ± 10.3		T: Bailing + ARB			
2	Feng et al. (2019)	C: 24	C:45.1 ± 5.2	CGN; DN; HN	C: KA	5 g, tid	12	24 hUP; BUN; Scr
		T: 23	T:44.6 ± 5.3		T: Bailing + KA			CRP; TNF-α; IL-6
3	Guo. (2022)	C: 28	C:38.47 ± 5.20	CGN	C: ARB	2.0 g, tid	24	BUN; Scr
		T: 27	T:39.10 ± 6.22		T: Bailing + ARB			CRP; TNF-α
4	He et al. (2018)	C: 31	C:48.13 ± 6.44	CGN	C: ARB	1.0 g, tid	12	24 hUP; BUN; Scr
		T: 34	T:47.69 ± 6.37		T: Bailing + ARB			
5	Huang et al. (2014)	C: 22	C:51.4 ± 11.24	DN(CKD III)	C: CMT	1.0 g, tid	12	CRP; TNF-α; IL-6
		T: 22	T:53.1 ± 12.34		T: Bailing + CMT			
6	Jia et al. (2021)	C: 23	C:50.29 ± 8.73	CGN; DN; HN; GN; PKD (CKD II∼III)	C: CMT	2.0 g, tid	12	BUN; Scr
		T: 22	T:49.53 ± 8.28		T: Bailing + CMT			CRP; TNF-α; IL-6
7	Li et al. (2019)	C: 23	C:338.7 ± 5.29	CGN	C: ACEI	1.0 g, tid	8	24 hUP; BUN; Scr
		T: 22	T:39.24 ± 4.86		T: Bailing + ACEI			
8	Liu (2021a)	C: 37	C:65.12 ± 5.89	CKD I∼III	C: ARB	1.5 g, tid	24	BUN; Scr
		T: 38	T:64.38 ± 6.15		T: Bailing + ARB			CRP; TNF-α
9	Liu and Liu (2015)	C: 36	C:43.1 ± 10.8	CGN	C: ARB	1.0 g, tid	16	24 hUP; BUN; Scr
		T: 39	T:41.7 ± 12.6		T: Bailing + ARB			
10	Liu et al. (2017)	C: 25	C:38.89 ± 3.87	CGN	C: alprostadil	2.5 g, tid	12	24 hUP
		T: 26	T:39.52 ± 4.10		T:Bailing + alprostadil			
11	Liu et al. (2017)	C: 31	C:43.14 ± 4.68	CGN	C: ARB	1.0 g, tid	16	BUN; Scr
		T: 28	T:43.22 ± 4.51		T: Bailing + ARB			
12	Liu. (2006)	C: 15	C:42.7	CGN; DN; HN (CKD I∼II)	C: ARB	1.0 g, tid	12	24hUP; Scr
		T: 18	T:41.3		T: Bailing + ARB			
13	Ma and Yan. (2021)	C: 103	C:43.61 ± 2.05	CGN	C: ARB	2.0 g, tid	12	24 hUP; BUN; Scr
		T: 97	T:43.29 ± 2.11		T: Bailing + ARB			
14	Qiu. (2019)	C: 17	C:52.38 ± 4.46	CGN; DN; HN	C:alprostadil	2.0 g, tid	12	24 hUP; BUN; Scr
		T: 18	T:52.41 ± 4.53		T:Bailing + alprostadil			CRP; TNF-α; IL-6
15	Quan et al. (2022)	C: 22	C:43.76 ± 7.92	CGN	C: ferulic	2.0 g, tid	4	24 hUP; BUN; Scr
		T: 20	T:43.29 ± 2.11		T: Bailing + ferulic			TNF-α; IL-6
16	Shao and Yu (2022)	C: 27	C:49.2 ± 3.9	CGN	C: ARB	2.0 g, tid	12	BUN; Scr
		T: 28	T:48.7 ± 4.1		T: Bailing + ARB			TNF-α
17	Song. (2019)	C: 30	C:44.2 ± 5.8	CGN	C: CCB	1.0 g∼3.0, tid	8	BUN; Scr
		T: 32	T:45.5 ± 3.7		T: Bailing + CCB			
18	Sun and Zhao (2021)	C: 55	C:50.08 ± 4.83	CGN	C: ARB	0.8 g, tid	12	24 hUP; BUN; Scr
		T: 53	T:50.01 ± 4.78		T: Bailing + ARB			
19	Tang and Xu. (2019)	C: 25	C:45.66 ± 11.44	CGN	C: ARB	1.0 g, tid	16	24 hUP; BUN; Scr
		T: 24	T:45.62 ± 12.13		T: Bailing + ARB			CRP; TNF-α; IL-6
20	Tang. (2022)	C: 13	C:45.33 ± 5.91	CGN	C: ARB	1.0 g, tid	10	BUN; Scr
		T: 13	T:45.21 ± 5.62		T: Bailing + ARB			
21	Wang et al. (2019)	C: 18	C:46.64 ± 6.04	CGN	C: ARB	2.0 g, tid	12	BUN; Scr
		T: 17	T:46.53 ± 6.02		T: Bailing + ARB			
22	Yang et al. (2009)	C: 19	C:31.6 ± 10.4	CGN; DN; LGN; DN; HSPN;	C: ARB	-	12	24 hUP; Scr
		T: 18	T:32.3 ± 11.5		T: Bailing + ARB			
23	Yu et al. (2019)	-	C:36.25 ± 11.69	CKD II∼III	C: ARB	2.0 g, tid	-	24 hUP; BUN; Scr
			T:34.85 ± 12.18		T: Bailing + ARB			CRP; TNF-α
24	Zhang. (2021a)	C: 20	C:48.65 ± 6.79	CKD I∼II	C: ARB	4.0 g, qd	12	24 hUP; BUN; Scr
		T: 25	T:47.43 ± 6.57		T: Bailing + ARB			
25	Zhang (2021b)	C: 29	C:45.62 ± 2.13	CGN	C: CTX	2.0 g, tid	12	BUN; Scr
		T: 28	T:45.65 ± 2.17		T:Bailing + CTX			TNF-α
26	Zhang (2022)	C: 17	C:42.61 ± 3.54	CGN	C: ARNI	2.0 g, tid	12	24 hUP; BUN; Scr
		T: 19	T:44.06 ± 5.18		T: Bailing + ARNI			
27	Zeng et al. (2018)	C: 20	C:60.51 ± 8.73	CGN; DN; HN (CKD II)	C: KA	2.0 g, tid	12	BUN; Scr
		T: 22	T:62.36 ± 8.91		T: Bailing + KA			CRP; TNF-α; IL-6
28	Zheng et al. (2019)	C: 32	C:52.52 ± 4.36	CKD III∼IV	C: CMT	1.5 g, tid	12	BUN; Scr
		T: 24	T:54.02 ± 3.21		T: Bailing + CMT			
29	Zhu et al. (2017)	C: 37	C:59.13 ± 5.36	CGN	C: ACEI	1.0 g, tid	24	24 hUP; BUN; Scr
		T: 39	T:59.87 ± 5.51		T: Bailing + ACEI			
30	Cui et al. (2023)	C: 48	C:72.43 ± 3.45	CGN	C:ARB	0.8 g, tid	12	24 hUP; BUN; Scr;
		T: 48	T:72.34 ± 3.42		T:Bailing + ARB			CRP; TNF-α
31	Hu. (2023)	C: 31	C:52.55 ± 4.91	CGN; DN; HN (CKD II)	C: alprostadil	2 g, tid	2	BUN; Scr
		T: 31	T:52.48 ± 4.86		T:Bailing + alprostadil			CRP; IL-6

CGN, chronic glomerulonephritis; DN, diabetic nephropathy; HN, hypertensive nephropathy; GN, gouty nephropathy; PKD, polycystic kidney disease; NS, nephrotic syndrome; LGN, latent glomerulopathy; LN, lupus nephritis; HSPN, henoch-schonlein purpura nephritis; LN, lupus nephriti; KA, compound alpha-ketoacid tablets; CMT, conventional medical treatments; CTX, cyclophosphamide.

### 3.2 Incorporate studies features

**FIGURE 2:**
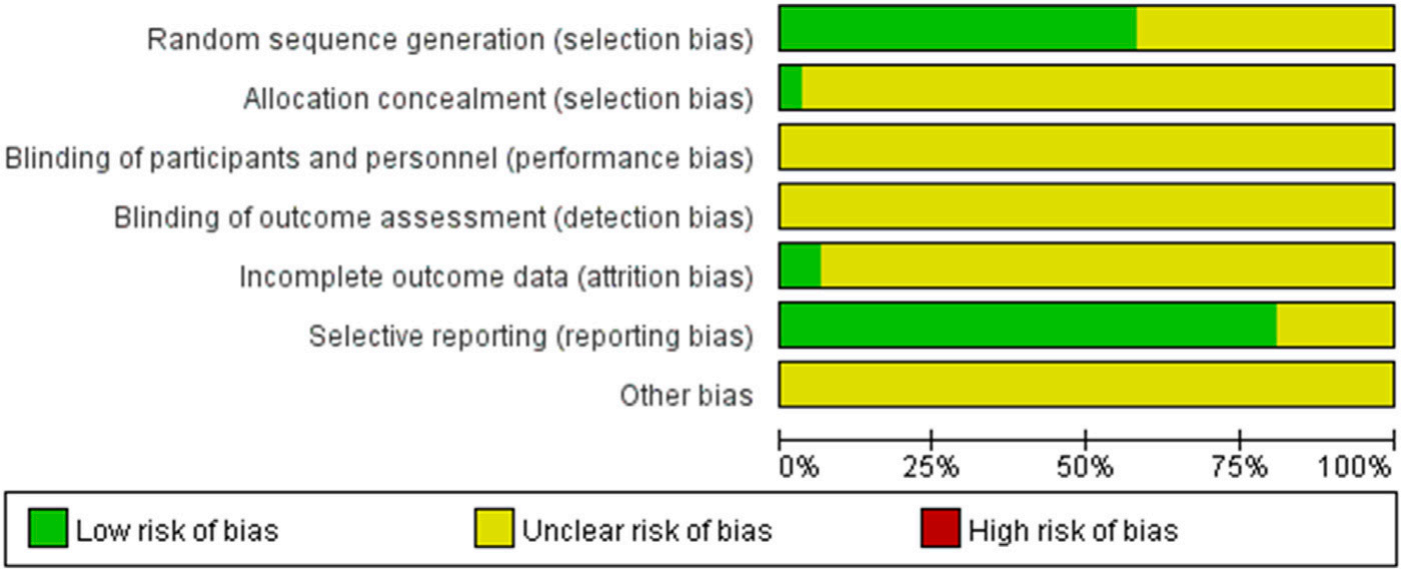
The risk of bias assessment graph of included studies.

A total of 2,934 patients were enrolled in the 31 studies, with 1,468 allocated to the control group and 1,466 to the experimental group. The quality assessment of the 31 included RCT articles was conducted using the Cochrane Collaboration Assessment Tool. The results of the quality assessment for bias analysis are presented in Figure 2.

### 3.3 Meta-analysis results

#### 3.3.1 Blood urea nitrogen (BUN) metrics

**FIGURE 3:**
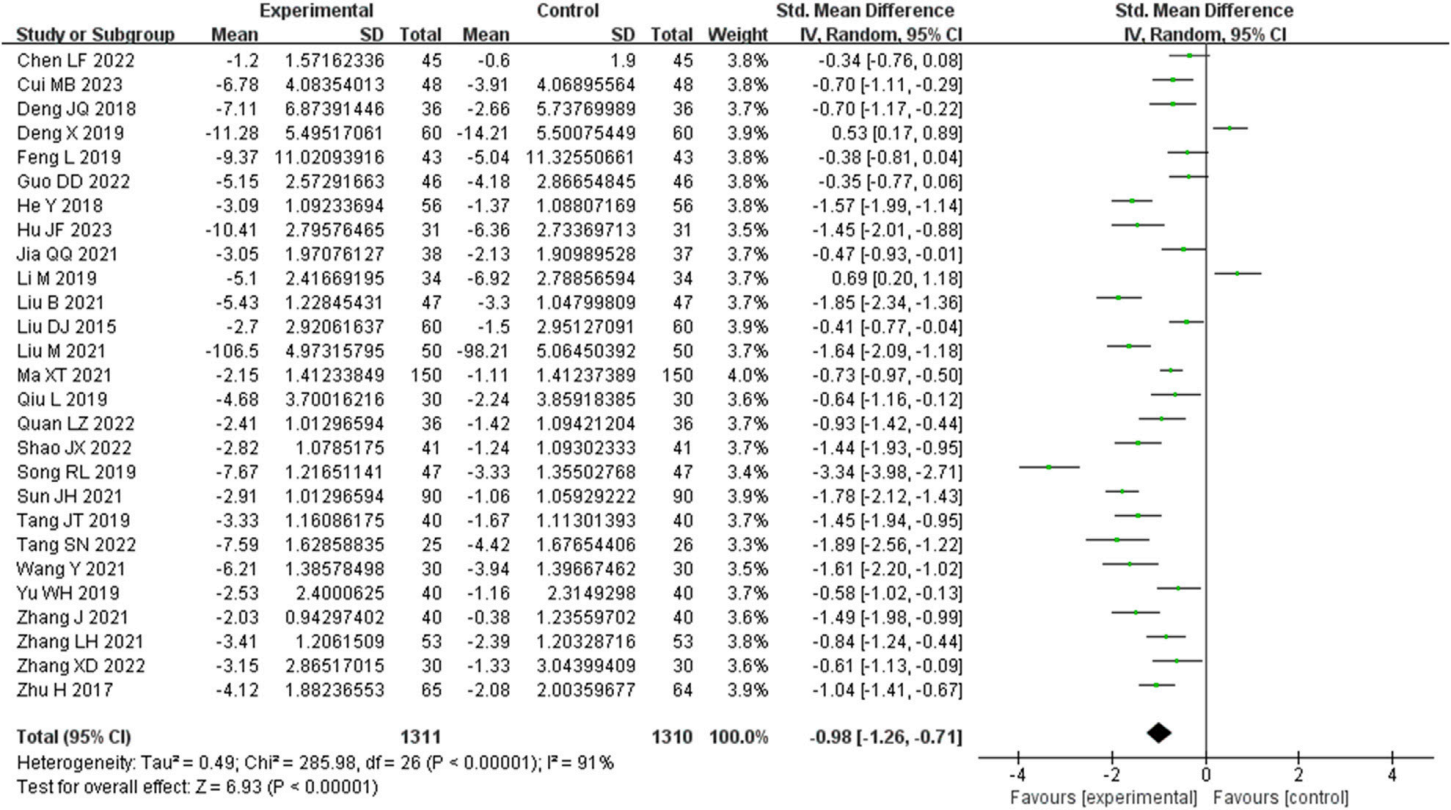
Comparative forest plots of BUN level. I^2^ and P were used as heterogeneity tests. Forest plot showing the effect of bailing on the outcome of blood urea nitrogen.

Twenty-seven studies analyzed BUN. There was high heterogeneity between studies (I^2^ = 91%), necessitating the use of a random-effects model to estimate the SMD. The results demonstrated that, when compared with the control group, the experimental group achieved a significant reduction in BUN levels (SMD = −0.98, 95% CI (−1.26, −0.71), *p* < 0.00001), and this difference held statistical significance (Figure 3). The analysis was further conducted based on the treatment duration. Among these 27 studies, there were 3 studies with a treatment duration of 24 weeks, 3 studies with a treatment duration of 16 weeks, 14 studies with a treatment duration of 12 weeks, and 3 studies with a treatment duration of 8 weeks. The remaining three studies were treated for 2, 4, and 10 weeks respectively, while one study did not specify the treatment duration. Because these groups contained only one included study, no analysis was performed. In 24 weeks treatment duration group, the BUN levels were significantly different (SMD = −1.07, 95% CI (−1.86, −0.28), *p* < 0.0001); In CGN, chronic glomerulonephritis; DN, diabetic nephropathy; HN, hypertensive nephropathy; GN, gouty nephropathy; PKD, polycystic kidney disease; NS, nephrotic syndrome; LGN, latent glomerulopathy; LN, lupus nephritis; HSPN, henoch-schonlein purpura nephritis; LN, lupus nephriti; KA, compound alpha-ketoacid tablets; CMT, conventional medical treatments; CTX, cyclophosphamide. 16 weeks treatment duration group, the BUN levels were significantly different (SMD = −1.15, 95% CI (−1.97, −0.34), *p* < 0.0001); In 12 weeks treatment duration group, the BUN levels were significantly different (SMD = −0.88, 95% CI (−1.22, −0.54), *p* < 0.00001); In 8 weeks treatment duration group, the difference was statistically significant (SMD = −0.99, 95% CI (−3.05, −1.08), *p* < 0.00001) (Supplementary Figure S1).

#### 3.3.2 Serum creatinine (SCR) metrics

**FIGURE 4:**
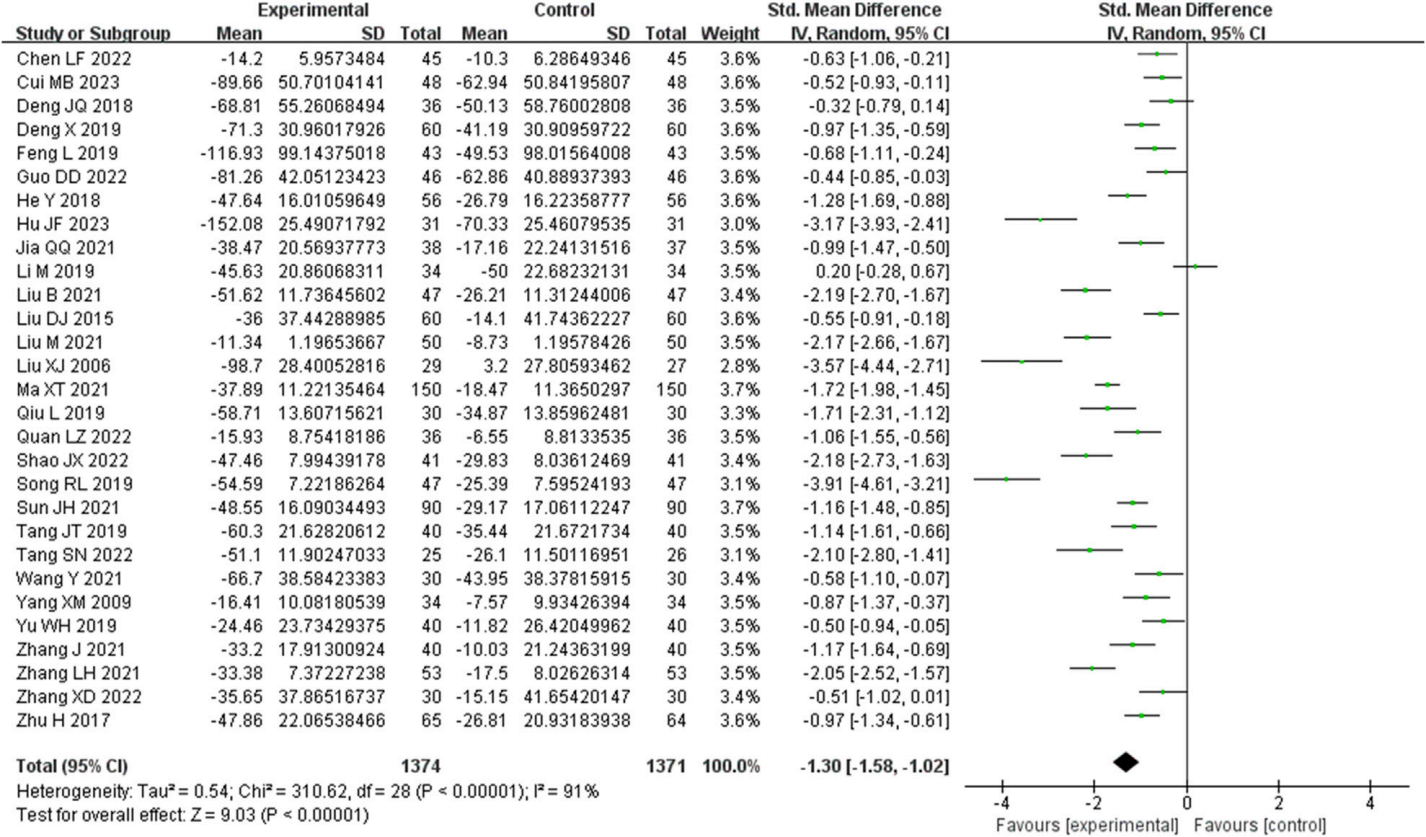
Comparative forest plots of Scr level. I2 and P were used as heterogeneity tests. Forest plot showing the effect of bailing on the outcome of Scr level.

Serum creatinine was examined in 29 studies. These studies exhibited a substantial degree of heterogeneity (I^2^ = 91%), necessitating the use of a random-effects model to estimate the SMD. The results indicated that, when compared with the control group, the experimental group achieved a significant reduction in Scr levels (SMD = −1.30, 95% CI (−1.58, −1.02), *p* < 0.00001), and this difference was statistically significant (Figure 4). The analysis was further conducted based on the treatment duration. The 29 studies included in this analysis comprised of 3 studies with a treatment duration of 24 weeks, 3 studies with a treatment duration of 16 weeks, 16 studies with a treatment duration of 12 weeks, and finally, 3 studies with a treatment duration of 8 weeks. The remaining three studies respectively were treated for 2, 4, and 10 weeks and one study did not mention treatment duration. In 24 weeks treatment duration group, the Scr levels were significantly different (SMD = −1.19, 95% CI (−2.10, −0.28), *p* < 0.00001); In 16 weeks treatment duration group, the Scr levels were significantly different (SMD = −1.27, 95% CI (−2.20, −0.34), *p* < 0.00001); In 12 weeks treatment duration group, the Scr levels were significantly different (SMD = −1.23, 95% CI (−1.54, −0.92), *p* < 0.00001); In 8 weeks treatment duration group, the difference was statistically significant (SMD = −1.43, 95% CI (−3.47, 0.61), *p* < 0.00001) (Supplementary Figure S2).

#### 3.3.3 24 h urinary protein (24 h UP) metrics

**FIGURE 5:**
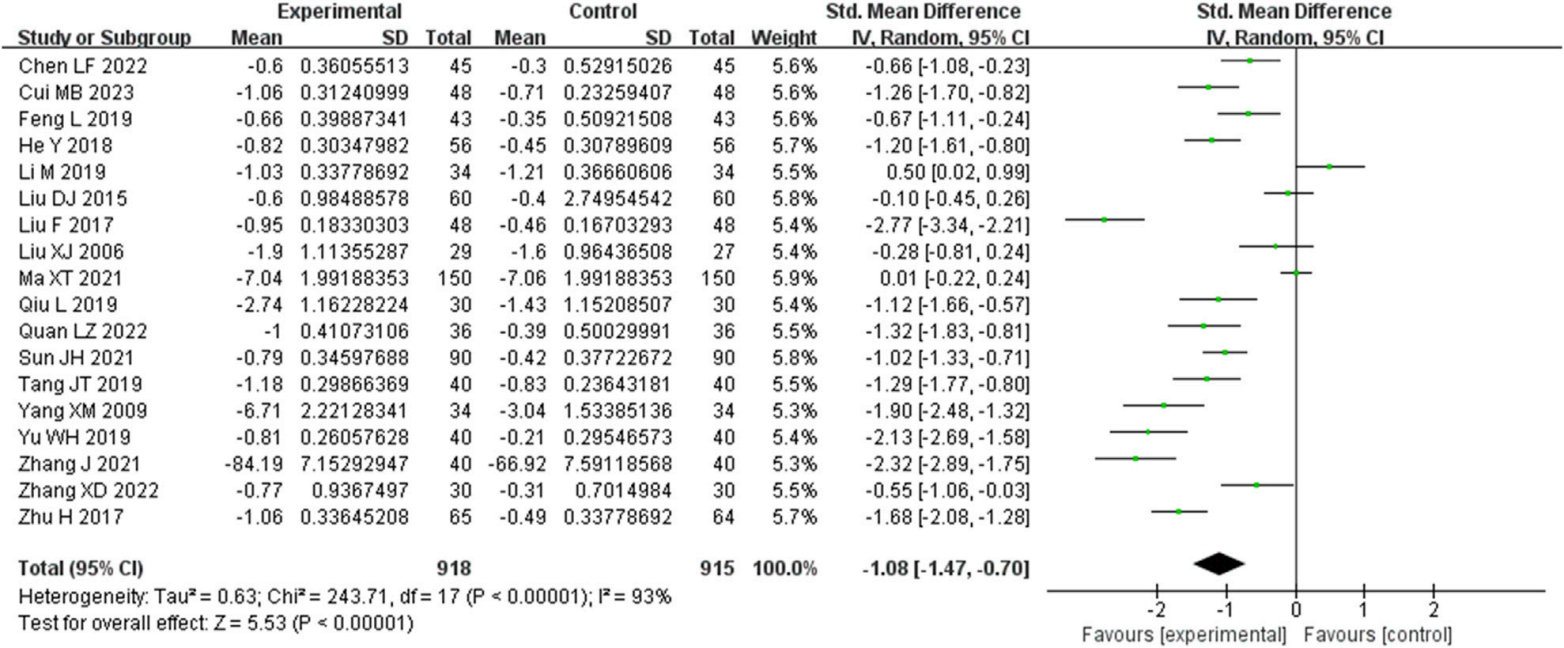
Comparative forest plots of 24hUP level. I2 and P were used as heterogeneity tests. Forest plot showing the effect of bailing on the outcome of 24hUP level.

Eighteen studies analyzed 24-h urinary protein. These studies demonstrated a significant degree of heterogeneity (I^2^ = 93%), necessitating the utilization of a random-effects model to estimate SMD. The results indicated that, in comparison to the control group, the experimental group achieved a substantial reduction in the 24-h urinary protein level (SMD = −1.08, 95% CI (−1.47, −0.70), *p* < 0.00001), with this difference being of statistical significance (Figure 5). The analysis was further conducted based on the treatment duration. Included in these 18 studies were was 1 study with 24 weeks treatment duration, 2 studies with 16 weeks treatment duration, 11 studies with 12 weeks treatment duration, and 2 studies with 8 weeks treatment duration. The remaining two studies respectively were treated for 4 weeks and no mention treatment duration. In 16 weeks treatment duration group, the 24UP levels were significantly different (SMD = −0.68, 95% CI (−1.85, 0.49), *p* = 0.0001); In 12 weeks treatment duration group, the 24UP levels were significantly different (SMD = −1.17, 95% CI (−1.67, −0.67), *p* < 0.00001); In 8 weeks treatment duration group, the difference was statistically significant (SMD = −0.08, 95% CI (−1.22, 1.06), *p* = 0.0004) (Supplementary Figure S3).

#### 3.3.4 High-sensitivity C-reactive protein (Hs-CRP) metrics

**FIGURE 6:**
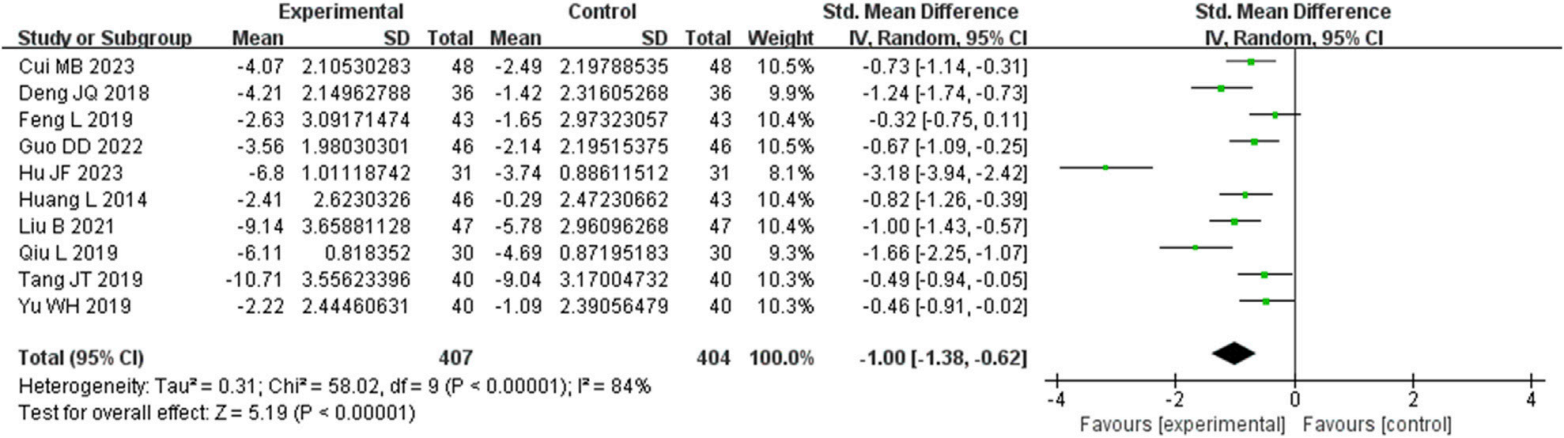
Comparative forest plots of Hs-CRP level. I^2^ and P were used as heterogeneity tests. Forest plot showing the effect of bailing on the outcome of Hs-CRP level.

Ten studies analyzed hs-CRP. These studies displayed heterogeneity (I^2^ = 84%), requiring the utilization of a random-effects model for estimating SMD. The results revealed that, in comparison to the control group, the experimental group achieved a significant reduction in the CRP level (SMD = −1.00, 95% CI (−1.38, −0.62), *p* = 0.004), with this difference being statistically significant (Figure 6). The analysis was further conducted based on the treatment duration. Included in these ten studies were 2 studies with 24 weeks treatment duration, 1 studies with 16 weeks treatment duration, 5 studies with 12 weeks treatment duration, and no studies with 8 weeks treatment duration. The remaining two studies were treated for 2 weeks and no mention treatment duration. In 24 weeks treatment duration group, the CRP levels were no significantly different (SMD = −0.83, 95% CI (−1.16, −0.51), *p* = 0.29); In 12 weeks treatment duration group, the CRP levels were significantly different (SMD = −0.92, 95% CI (−1.34, −0.51), *p* = 0.003) (Supplementary Figure S4).

#### 3.3.5 Tumor necrosis factor-α (TNF-α) metrics

**FIGURE 7:**
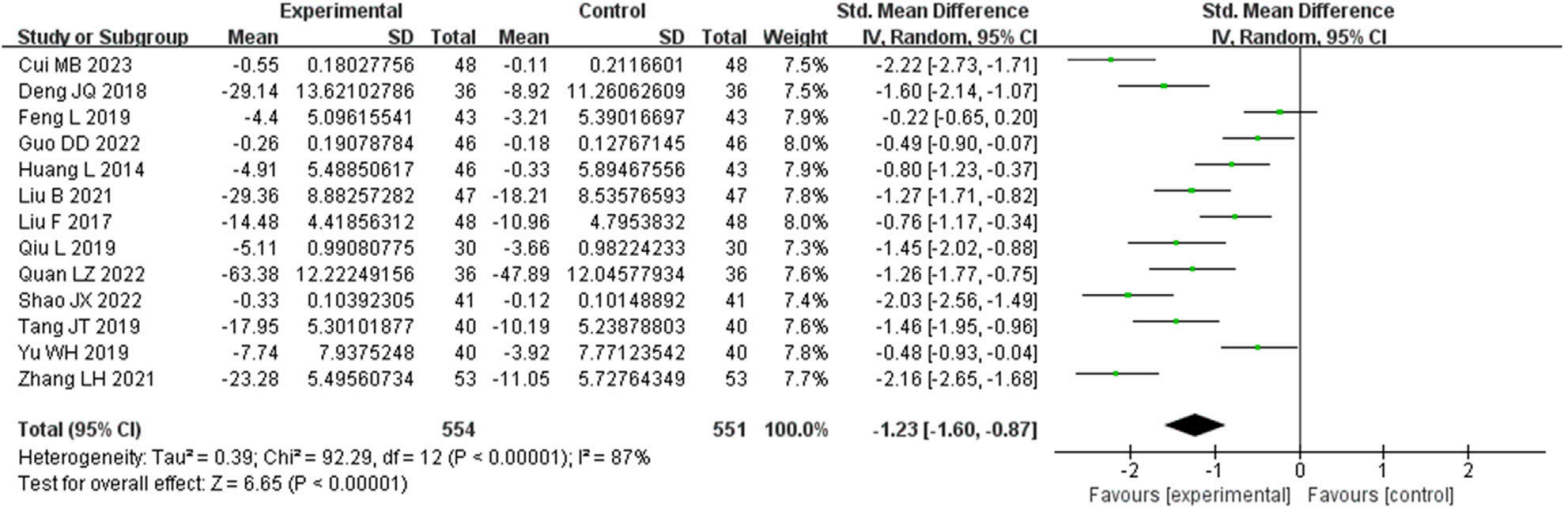
Comparative forest plots of TNF-α level. I2 and P were used as heterogeneity tests. Forest plot showing the effect of bailing on the outcome of TNF-α level.

Thirteen studies assessed TNF-α levels. These studies displayed a notable degree of heterogeneity (I^2^ = 87%), necessitating the application of a random-effects model to estimate SMD. The findings indicated that, when compared with the control group, the experimental group achieved a significant reduction in TNF-α levels (SMD = −1.23, 95% CI (−1.60, −0.87), *p* < 0.00001), with this difference being statistically significant (Figure 7). The analysis was further conducted based on the treatment duration. Included in these thirteen studies were 2 studies with 24 weeks treatment duration, 1 studies with 16 weeks treatment duration, 8 studies with 12 weeks treatment duration, and no studies with 8 weeks treatment duration. The remaining two studies were treated for 4 weeks and no mention treatment duration. In 24 weeks treatment duration group, the TNF-α levels were significantly different (SMD = −0.87, 95% CI (−1.64, −0.11), *p* = 0.01); In 12 weeks treatment duration group, the TNF-α levels were significantly different (SMD = −1.39, 95% CI (−1.93, −0.86), *p* < 0.00001) (Supplementary Figure S5).

#### 3.3.6 Interleukin-6 (IL-6) metrics

**FIGURE 8:**
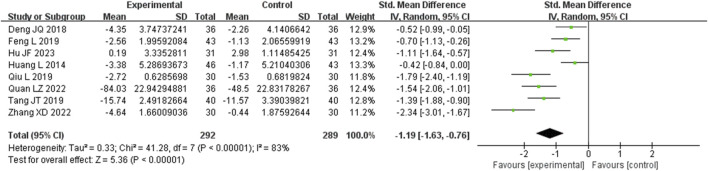
Comparative forest plots of IL-6 level. I^2^ and P were used as heterogeneity tests. Forest plot showing the effect of bailing on the outcome of IL-6 level.

Eight studies assessed IL-6 levels. These studies displayed a substantial degree of heterogeneity (I^2^ = 83%), necessitating the use of a random-effects model to estimate the SMD. The results demonstrated that, when compared with the control group, the experimental group significantly reduced IL-6 levels (SMD = −1.19, 95% CI (−1.63, −0.76), *p* < 0.00001), with this difference achieving statistical significance (Figure 8). The analysis was further conducted based on the treatment duration. Included in these eight studies were no studies with 24 weeks treatment duration, 1 studies with 16 weeks treatment duration, 5 studies with 12 weeks treatment duration, and no studies with 8 weeks treatment duration. The remaining two studies were treated for 4 and 2 weeks. In 12 weeks treatment duration group, the IL-6 levels were significantly different (SMD = −1.12, 95% CI (−1.78, −0.45), *p* < 0.00001) (Supplementary Figure S6).

#### 3.3.7 Sensitivity analysis and publication bias assessment

After a meticulous examination with a step-by-step removal of articles and subsequent sensitivity analysis, the findings remained robust, exhibiting no significant change. Furthermore, the results of the Egger’s regression and Begg’s rank correlation tests indicated the presence of publication bias for all indicators (*p* < 0.05) (Table 2).

**TABLE 2:** Publication bias evaluation of meta-analysis of Bailing capsule treatment.

Index	Begg		Egger	
	*z*	*P*	*t*	*P*
Scr	2.38	0.017	−2.13	0.043
24hUP	2.12	0.034	−3.11	0.007
BUN	2.13	0.033	−1.77	0.089
TNF-α	2.26	0.024	−3.90	0.002
hs-CRP	1.79	0.074	−6.49	0.000
IL-6	2.60	0.009	−5.87	0.001

### 3.4 Network pharmacology results of bailing for CKD

#### 3.4.1 Ingredients and target screening

We conducted a comprehensive search in the TCMSP database for Bailing Capsules (Cordyceps Sinensis) and identified a total of 38 active pharmaceutical ingredients. These ingredients were then screened based on OB ≥ 30% and DL ≥ 0.18, resulting in the identification of 7 major active components, namely arachidonic acid, Linoleyl acetate, beta-sitosterol, Peroxyergosterol, cerevisterol, cholesteryl palmitate, and CLR. Furthermore, the TCMSP database facilitated the prediction of target proteins associated with these active ingredients. Subsequently, we utilized the Unitprot database to query the gene names of these targets, eliminating any invalid or duplicated entries, ultimately culminating in the identification of 282 target genes.

#### 3.4.2 Disease target screening

The target proteins associated with CKD were obtained through a thorough search in the GeneCards database. After eliminating duplicate targets, a total of 4,368 CKD-related targets were obtained.

#### 3.4.3 Protein-protein interaction (PPI) result

**FIGURE 9:**
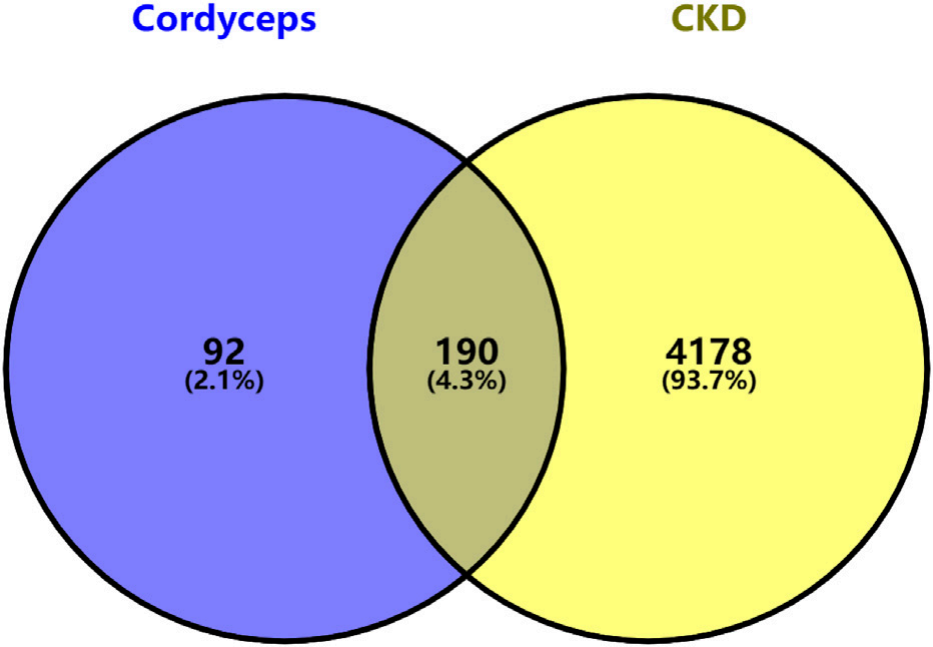
Venn diagram summarizing the intersection targets of cordyceps and CKD.

**FIGURE 10:**
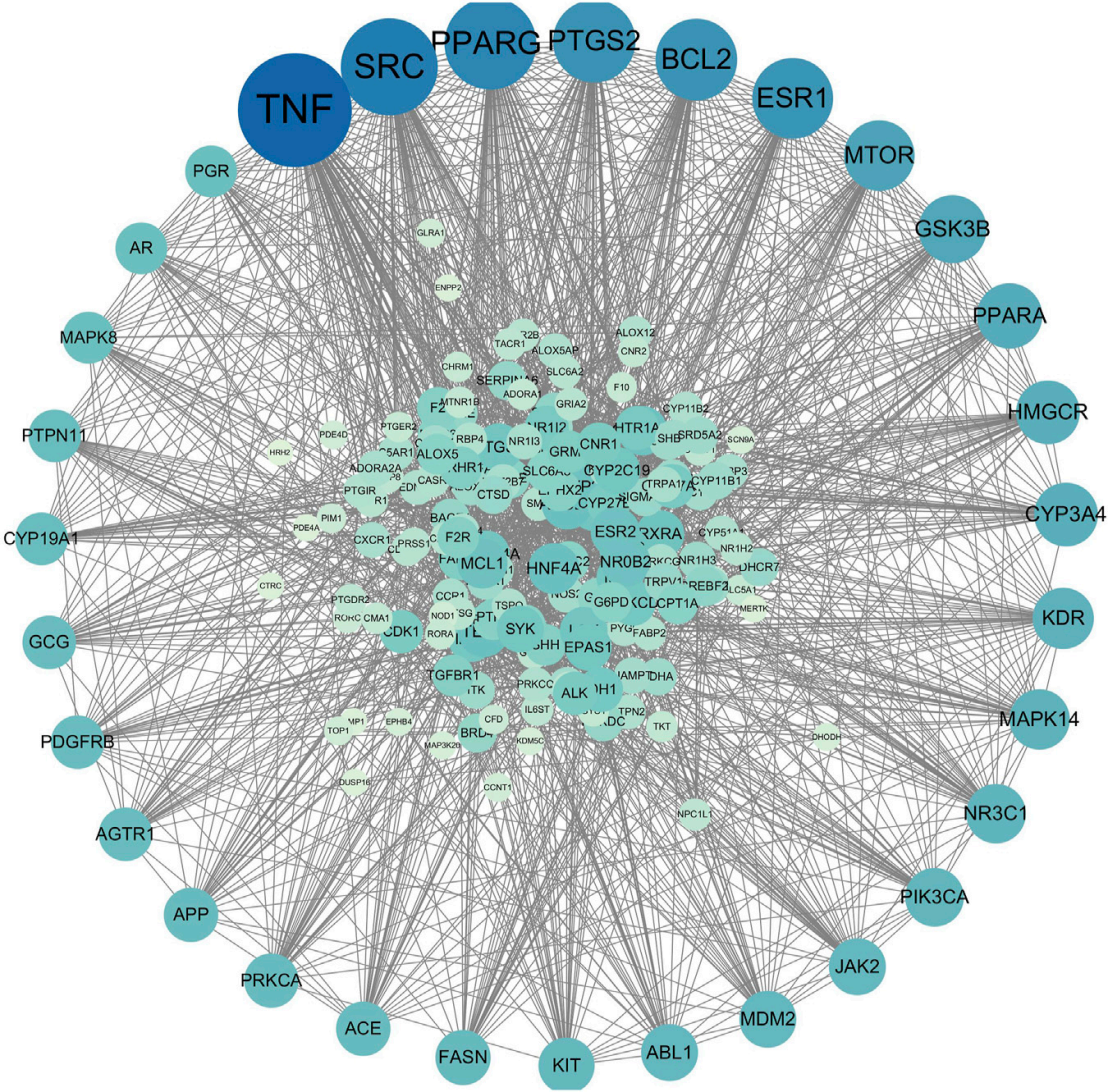
Protein-protein interaction network of treating CKD.

After aligning the obtained component targets of Bailing Capsule (Cordyceps sinensis) with the CKD-related targets, we identified 190 common targets (Figure 9). These common targets were subsequently subjected to analysis in the String Protein Interaction Network database, resulting in the construction of a PPI network model with a minimum interaction score of 0.9 (Figure 10). This network comprised a total of 189 nodes connected by 1,782 edges. Notably, the top 31 targets, which included TNF, SRC, PPARG, PTGS2, BCL2, ESR1, MTOR, GSK3B, PPARA, CYP3A4, HMGCR, KDR, MAPK14, NR3C1, PIK3CA, JAK2, ABL1, MAM2, KIT, FASN, ACE, APP, PRKCA, AGTR1, GCG, PDGFRB, CYP19A1, PTPN11, PGR, AR, and MAPK8, exhibited the high degree values. As depicted in the figure, TNF had the highest degree with 104, followed by SRC with 84, PPARG with 78, PTGS2 with 67, BCL2 with 66, ESR1 with 65, MTOR with 53, GSK3B with 50, PPARA with 47, CYP3A4 with 45, HMGCR with 45, KDR with 42, MAPK14 with 41, NR3C1 with 40, PIK3CA with 39, JAK2 with 37, ABL1 with 37, MAM2 with 37, KIT with 36, FASN with 35, ACE with 34, APP with 34, PRKCA with 34, AGTR1 with 33, GCG with 33, PDGFRB with 33, CYP19A1 with 32, PTPN11 with 32, PGR with 31, AR with 31, and MAPK8 with 31.

#### 3.4.4 Constrution and analysis of “ingredient-target-disease relationship network”

**FIGURE 11:**
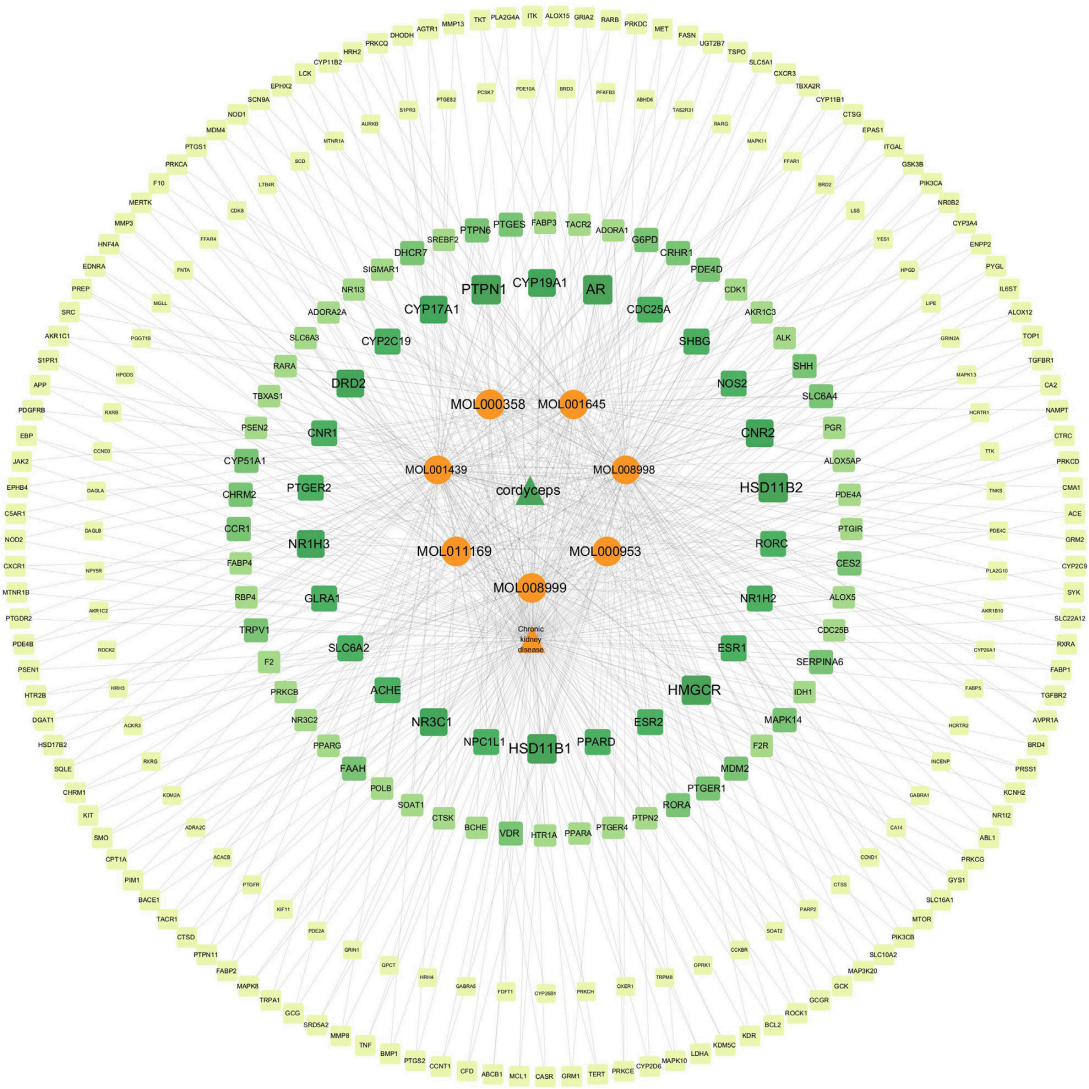
Ingredient-target-disease relationship network.

We imported the active drug ingredients, the CKD-related targets, and the drug-related targets into Cytoscape software, and constructed an ingredient-target-disease relationship network of Cordyceps sinensis and CKD (Figure 11). This network included a total of 289 nodes and 666 edges, providing a comprehensive illustration of Cordyceps sinensis treatment in CKD, characterized by its multi-component and multi-target features. Notably, targets with a degree value above 5 included PTPN1, HSD11B1, HSD11B2, HMGCR, AR, NR1H3, NR3C1, CNR2, CYP19A1, CYP17A1, and DRD2. Among the active ingredients of Cordyceps sinensis, those with degree values above 70 were MOL008998 (cerevisterol), MOL001439 (arachidonic acid), and MOL001645 (Linoleyl acetate), suggesting that cerevisterol, arachidonic acid, and Linoleyl acetate may play crucial roles in the effects of Cordyceps sinensis in treating CKD.

#### 3.4.5 GO bioanalysis and KEGG enrichment analysis

**FIGURE 12:**
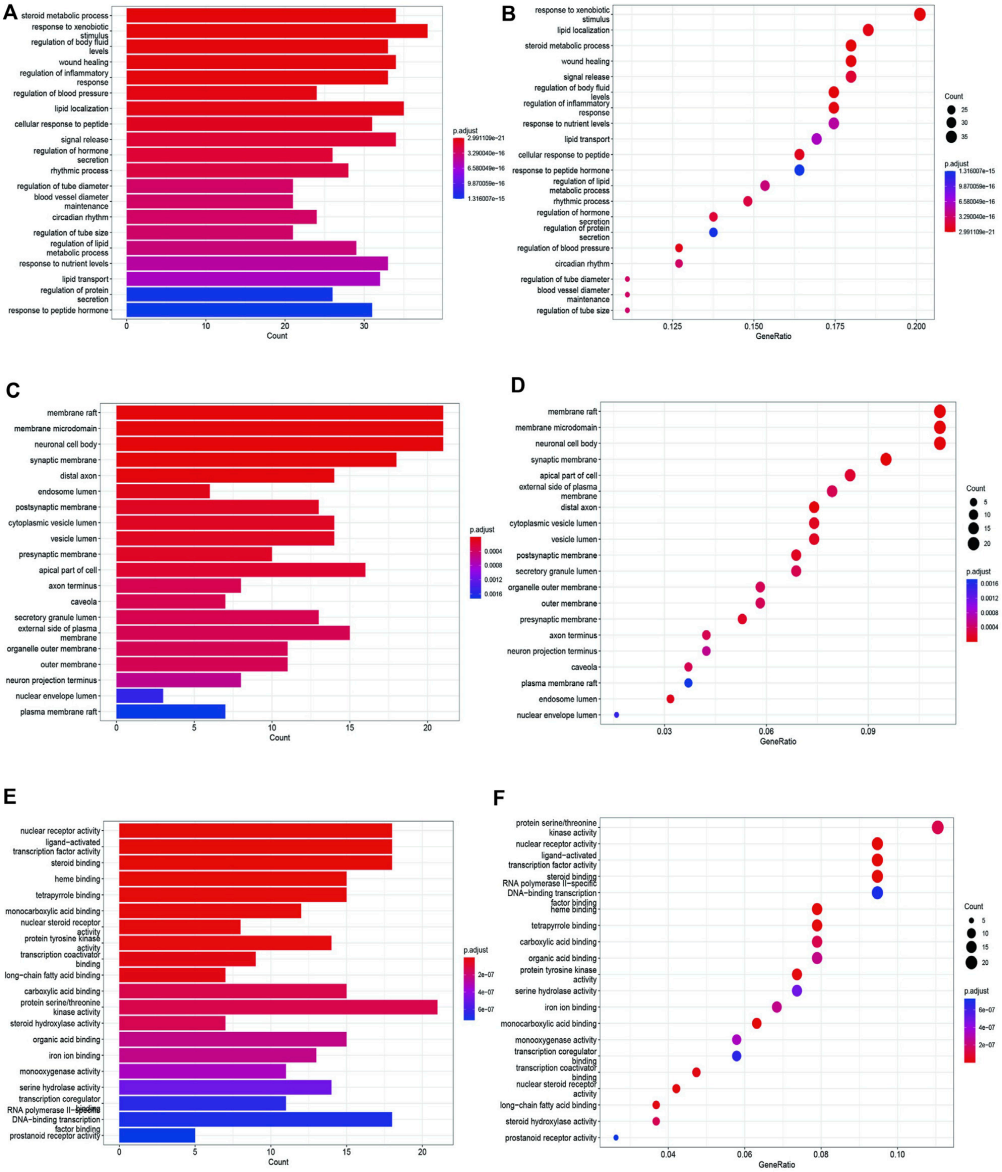
GO enrichment analysis of treating CKD targets. **(A,B)** GO-BP analysis, **(C,D)** GO-CC analysis, **(E,F)** GO-MF analysis.

**FIGURE 13:**
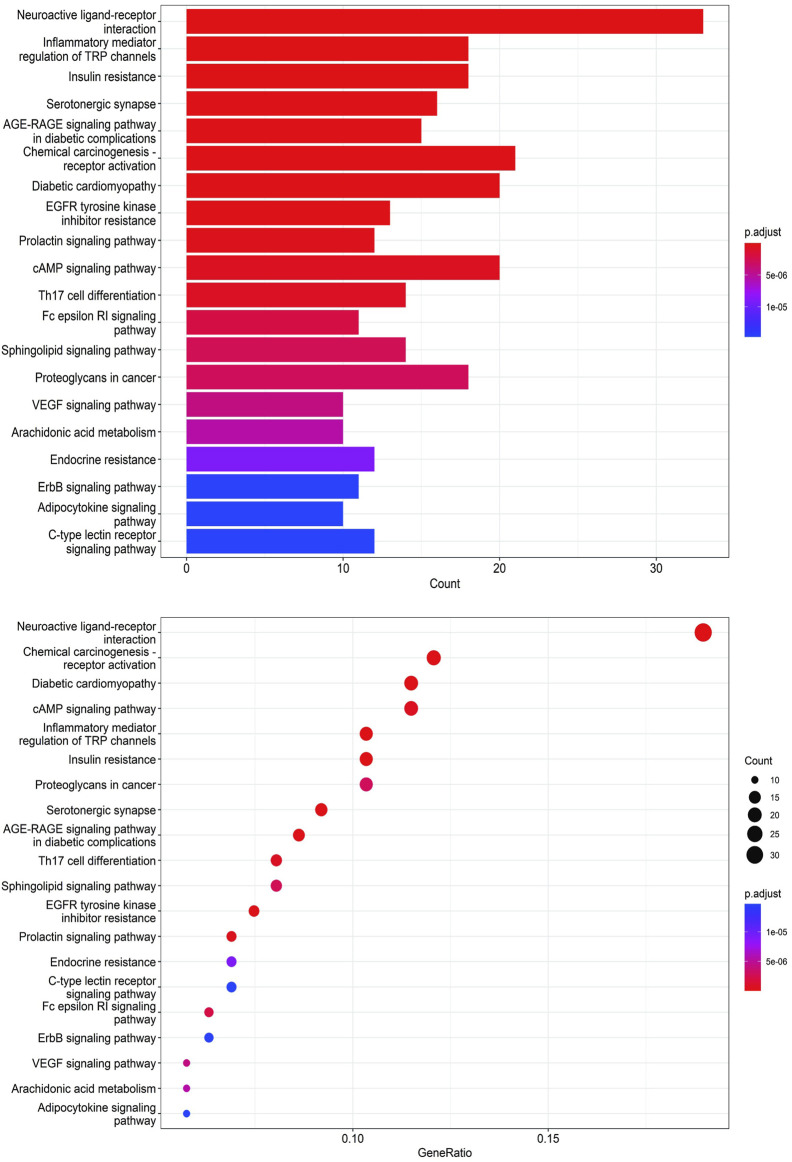
KEGG enrichment analysis of treating CKD targets.

To unravel the intricate mechanism of Bailing Capsule (Cordyceps Sinensis) in the treatment of CKD, we conducted GO analyses for Biological Processes (BP), Cellular Components (CC), and Molecular Functions (MF) based on the 190 common targets. Additionally, we performed KEGG enrichment analysis to gain deeper insights into the potential mechanisms of Bailing Capsule (Cordyceps sinensis) in CKD treatment. The GO enrichment analysis unveiled the top 20 most enriched terms in the BP, MF, and CC categories. Within the BP category, the target proteins were predominantly associated with lipid localization and steroid metabolic processes, among others. In the MF category, the target proteins were primarily linked to nuclear receptor activity and ligand-activated transcription factor activity, to name a few. In the CC category, the target proteins were primarily situated in locations such as membrane rafts, membrane microdomains, and neuronal cell bodies (Figure 12). A total of 134 pathways were identified in the KEGG pathway analysis. Figure 13 illustrates the top 20 matched KEGG pathways. The top 10 significant signaling pathways, as determined by p-values, included Neuroactive ligand-receptor interaction, Chemical carcinogenesis receptor activation, Diabetic cardiomyopathy, cAMP signaling pathway, Inflammatory mediator regulation of TRP channels, Insulin resistance, Proteoglycans in cancer, Serotonergic synapse, AGE-RAGE signaling pathway, EGFR tyrosine kinase inhibitor resistance, Prolactin signaling pathway, Endocrine resistance, C-type lectin receptor signaling pathway, ErbB signaling pathway, VEGF signaling pathway, Arachidonic acid metabolism, and Adipocytokine signaling pathway. These findings suggest that Cordyceps may influence the treatment of CKD by modulating key targets within these signaling pathways, with many therapeutic targets participating in multiple signaling pathways.

## 4 Discussion

CKD is a common condition in which kidney function gradually deteriorates. It is defined as kidney damage, indicated by markers such as abnormal urine or blood tests, or imaging, or a decrease in the GFR to less than 60 mL/min/1.73 m^2^, persisting for 3 months or more (Levey et al., 2003; Sun and Zhao, 2021). The causes of CKD are complex and diverse. Diabetes and hypertension are the leading causes of CKD in both developed and many developing countries. Primary glomerulonephritis, environmental pollution, pesticide exposure, analgesic abuse, herbal medicines, and the use of unregulated food additives also contribute to the burden of CKD in developing countries. Infectious diseases, such as hepatitis B, and genetic factors also play a role (Tang and Xu, 2019). Furthermore, the progression of CKD may be caused or exacerbated by underlying renal conditions, arterial hypertension, dyslipidemia, anemia, proteinuria, calcium and phosphate imbalances, as well as smoking (Sun and Zhao, 2021). When the renal function of CKD patients continues to deteriorate and reaches ESRD, renal replacement therapy (dialysis or kidney transplantation) is required. Additionally, adverse reactions, such as renal anemia, renal malnutrition, renal bone disease, etc., may occur and reduce the quality of life of CKD patients (Sun and Zhao, 2021). Therefore, delaying the progression of CKD and even reversing renal damage is a topic that we have been studying and discussing.

In China, Cordyceps sinensis, a traditional Chinese medicine known for its rich pharmacological potential, including immunomodulation, anti-inflammatory, antiviral, and antioxidant properties, is widely used in clinical practice to treat respiratory diseases, immune disorders, kidney diseases, and even various types of tumors (Yang et al., 2009; Wang, 2021; Tang, 2022). Bailing Capsules contain Cordyceps sinensis as the active ingredient and are one of the most commonly used traditional Chinese medicine ingredients for CKD patients. Our study integrated a substantial number of randomized controlled trials on Bailing Capsules, whether used alone or in combination, to treat CKD (with combination treatments being more prevalent). The results indicate that Bailing Capsules effectively reduce BUN, Scr, and 24hUP levels in CKD patients. Additionally, Bailing Capsules significantly lower the levels of inflammatory factors such as hs-CRP, TNF-α, and interleukin-6 IL-6. Among the randomized controlled studies we included, most involved Bailing Capsules combined with ACEI/ARB/ARNI, while some used Bailing Capsules in combination with ketoacid tablets, alprostadil, or conventional treatment. Regardless of the specific combination, the addition of Bailing Capsules effectively improved renal function indicators and reduced inflammatory markers in CKD patients. In summary, our meta-analysis demonstrates that Bailing Capsules can enhance the therapeutic effects of conventional CKD drug treatments, resulting in improved renal function and reduced inflammatory indicators.

Modern pharmacological research has provided valuable insights into the basic mechanisms through which Cordyceps sinensis treats CKD. Given the increasing prevalence of diabetic nephropathy as a cause of CKD, Cordyceps sinensis demonstrates its potential in improving renal function and urinary protein levels, reducing glomerulosclerosis, renal interstitial damage, and fibrosis in diabetic nephropathy animal models. This effect may be linked to the inhibition of the P2X7R/NLRP3 inflammasome expression and the regulation of the PPARα pathway (Yu et al., 2019; Zhang, 2021a). Additionally, Cordyceps sinensis has the potential to mitigate renal damage and the associated inflammatory response by targeting the TGF-β1/Smad and TLR4/NF-κB signaling pathways in diabetic nephropathy animal models (Zhang, 2021b). The protective role of Cordyceps sinensis is further substantiated through its ability to regulate processes like autophagy, apoptosis, and oxidative stress (Zhu et al., 2017; Zheng et al., 2019; Zhang, 2022). For hypertensive nephropathy, studies indicate that Cordyceps sinensis may alleviate renal tubular epithelial cell damage and tubulointerstitial fibrosis induced by hypertension. It was observed that Cordyceps sinensis can influence mitochondrial function and autophagy in both *in vivo* and *in vitro* experiments (National Kidney, 2002; Jha et al., 2013). In China, primary glomerular diseases account for a significant proportion of CKD cases. It have found that Cordyceps sinensis significantly reduces 24-h urinary protein, blood urea nitrogen, serum creatinine levels, and inflammatory markers in rats with membranous glomerulonephritis while increasing serum albumin and total serum protein levels. These effects were accompanied by enhanced activities of AKT and NF-κB p65 in the kidneys of rats with membranous glomerulonephritis (Das et al., 2020). In cases of IgA nephropathy, Cordyceps sinensis may curb the inflammatory response by regulating Th22 cell chemotaxis (Buenz et al., 2005). As CKD progresses, renal fibrosis contributes to worsening kidney damage and the development of ESRD. Cordyceps sinensis demonstrates effectiveness in addressing renal fibrosis. Using a rat model of unilateral ureteral obstruction (UUO), researchers observed that Cordyceps sinensis can inhibit BAG3 expression, thus reducing renal fibrosis (Liu et al., 2022). The inhibition of the TGF-β1/Smad classic signaling pathway and the reversal of epithelial-mesenchymal transition are among the mechanisms through which Cordyceps sinensis exerts its anti-renal fibrosis effects (Wang et al., 2018; Yang et al., 2020; Zhang et al., 2022). Cordyceps sinensis also exhibits inhibitory effects on the proliferation of human glomerular mesangial cells *in vitro* (Chen et al., 2019).

We used network pharmacology to further explore the mechanism of Cordyceps sinensis in treating CKD. Our findings reveal neuroactive ligand-receptor interactions, chemical oncogenic receptor activation, diabetic cardiomyopathy, cAMP signaling pathway, inflammatory mediator regulation of TRP channels, insulin resistance, proteoglycans in cancer, serotonin Energy synapse, AGE-RAGE signaling pathway, EGFR tyrosine kinase inhibitor resistance, and prolactin signaling pathway appeared both in the top 10 important signaling pathways in GO analysis and KEGG analysis. It indicates that Cordyceps sinensis can regulate the body’s immune response, control inflammation, regulate cell apoptosis, and improve vascular endothelial damage and tissue fibrosis through multiple pathways. From the construction of PPI and component-disease-target network diagram, it can be seen that Cordyceps sinensis regulates CKD immune response, oxidative stress and inflammatory response through multiple targets, thereby exerting a therapeutic effect. For the parts that have not yet been studied, we can further verify and explore through *in vivo* and *in vitro* experiments. (Zhao-Long et al., 2000; Pan et al., 2013; Du et al., 2015; Song et al., 2016; Takakura et al., 2017; Xiao et al., 2018; Zheng et al., 2018; Wang et al., 2019; Cai et al., 2021; Zhang et al., 2023a; Zhang et al., 2023b).

This analysis still has some limitations. Although a large number of studies were included in the meta-analysis, more rigorous clinical trials are still needed to provide more qualified evidence. The quality of included studies was compromised by a lack of detailed reporting on distribution concealment, randomization, and blinding. In network pharmacology, the Bailing capsule are a preparation made from cordyceps. Strictly, the chemical composition of Bailing capsules may not be exactly equivalent to cordyceps sinensis. Bailing capsule contains Cordyceps polysaccharides and amino acids or others, so the analysis may be interfered. And due to database limitations, drug active ingredients and corresponding targets, and some pathways have not been fully predicted, so a large number of experiments are needed to verify the prediction results.

In conclusion, Cordyceps sinensis has demonstrated its efficacy in the treatment of CKD. Our meta-analysis has clarified the impact of Bailing Capsules, which contain Cordyceps sinensis, on renal function and inflammatory factors in CKD patients. Furthermore, our network pharmacology analysis has provided insights into the active ingredients, therapeutic targets, and pathways through which Cordyceps sinensis exerts its effects, offering valuable evidence for the application of traditional Chinese medicine in CKD treatment. While our study has certain limitations, it paves the way for future research and the application of Cordyceps sinensis in CKD treatment.
