# Supplementary figures and images for: Use of bailing capsules (cordyceps sinensis) in the treatment of chronic kidney disease: a meta-analysis and network pharmacology
Source: Front Pharmacol. 2024 Apr 5;15:1342831. doi: 10.3389/fphar.2024.1342831 (PMC11026558; doi:10.3389/fphar.2024.1342831)

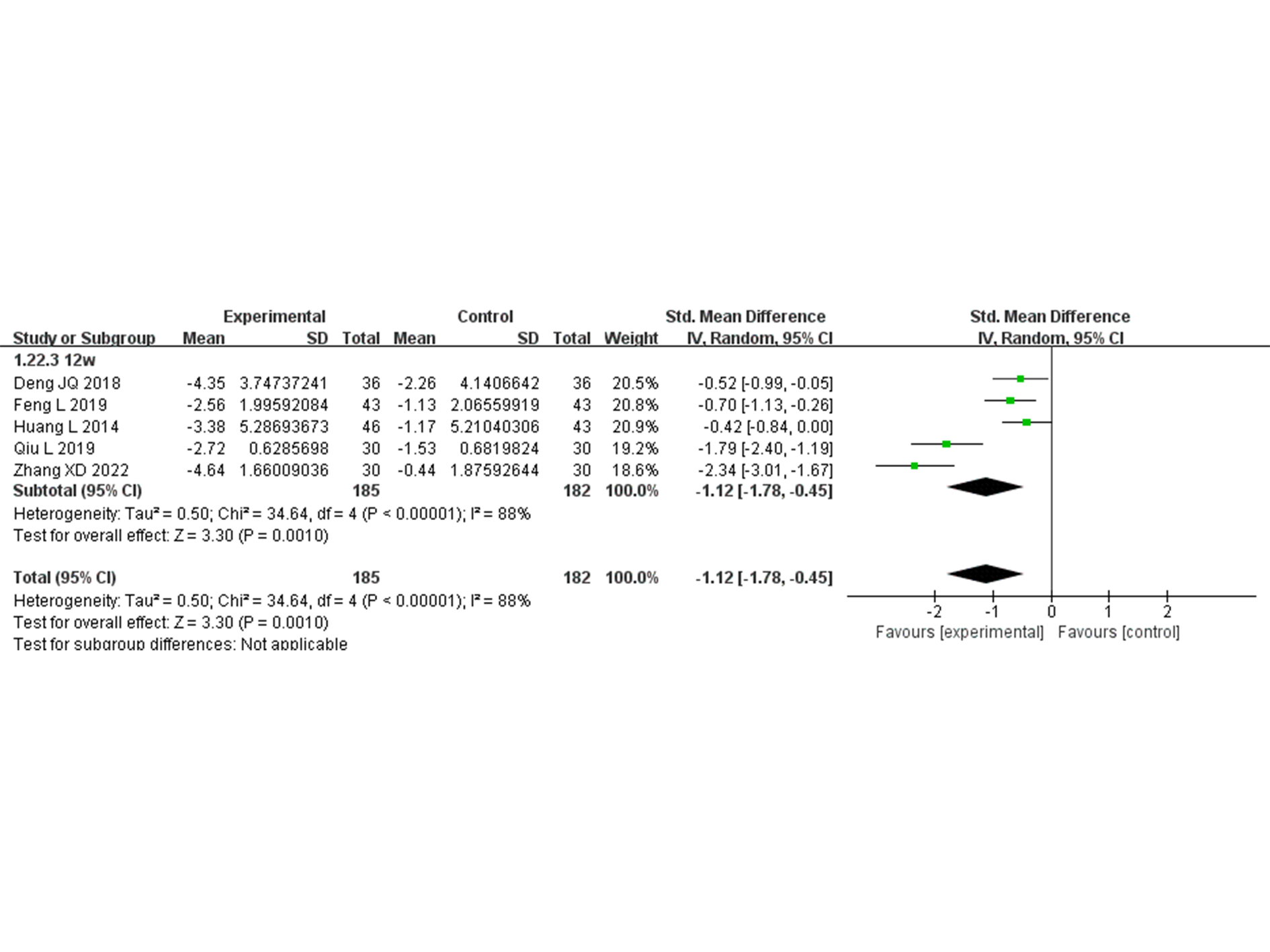

Supplement: Supplementary file 1 [file Image6.TIF]

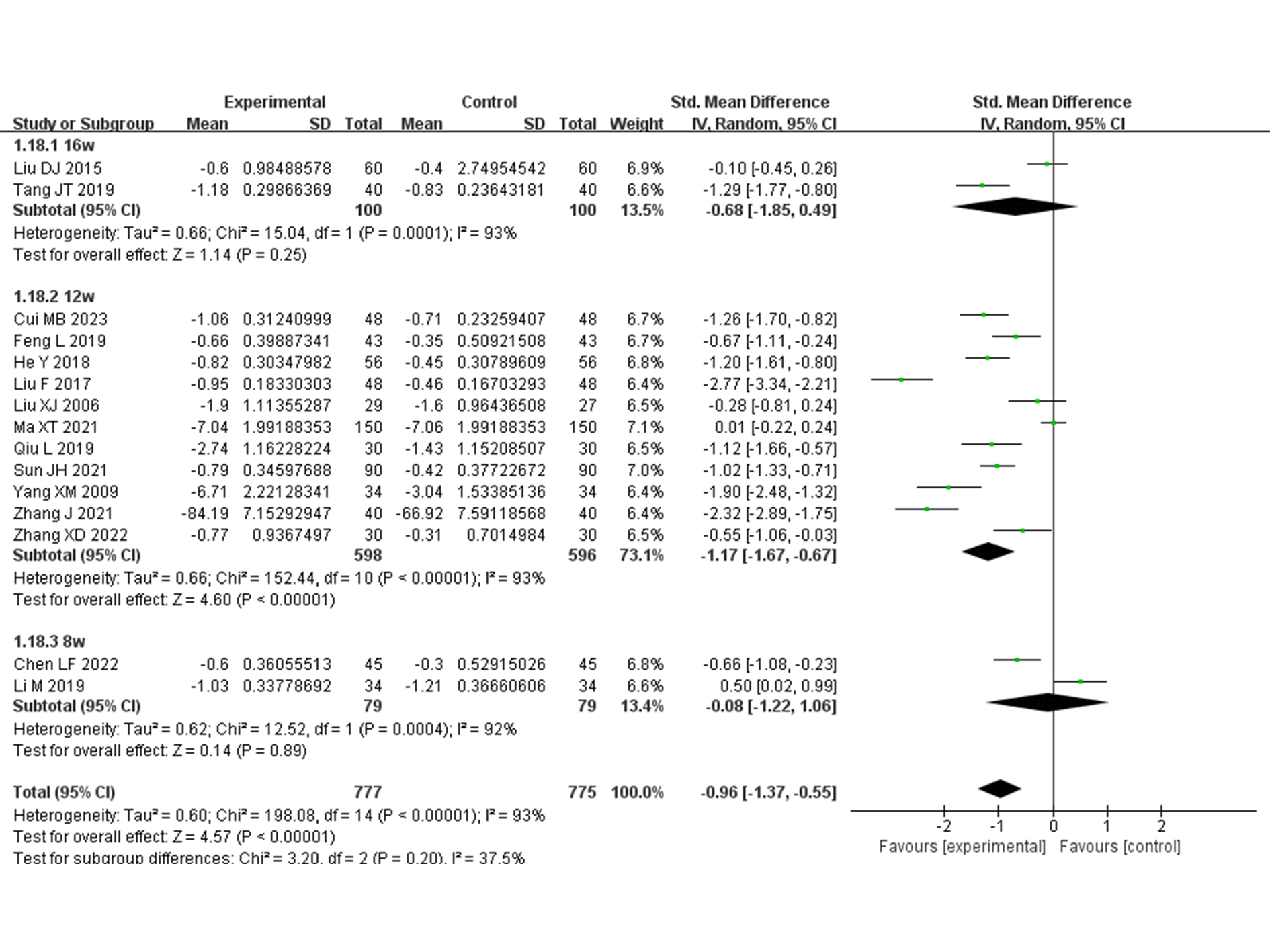

Supplement: Supplementary file 3 [file Image3.TIF]

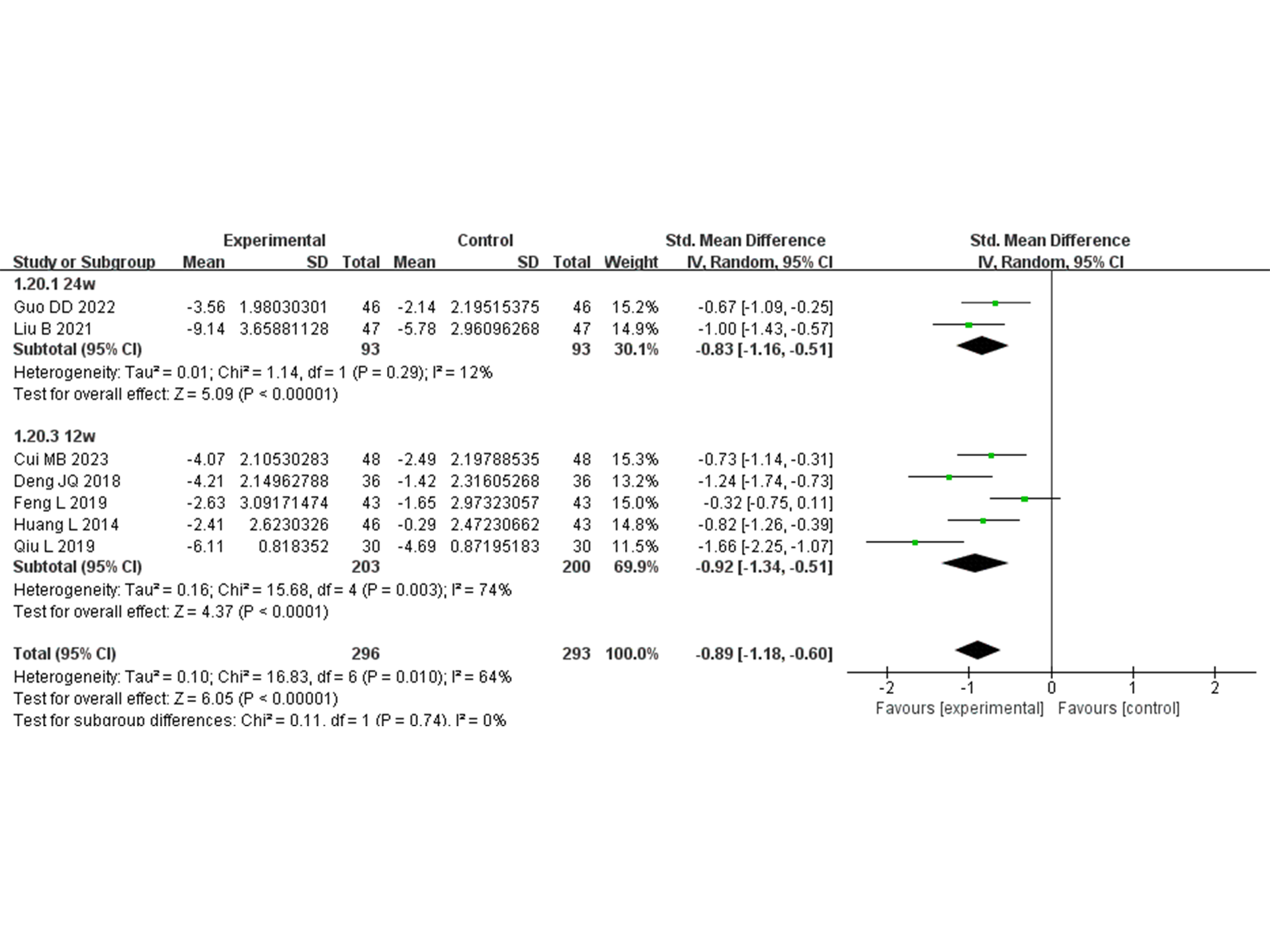

Supplement: Supplementary file 4 [file Image4.TIF]

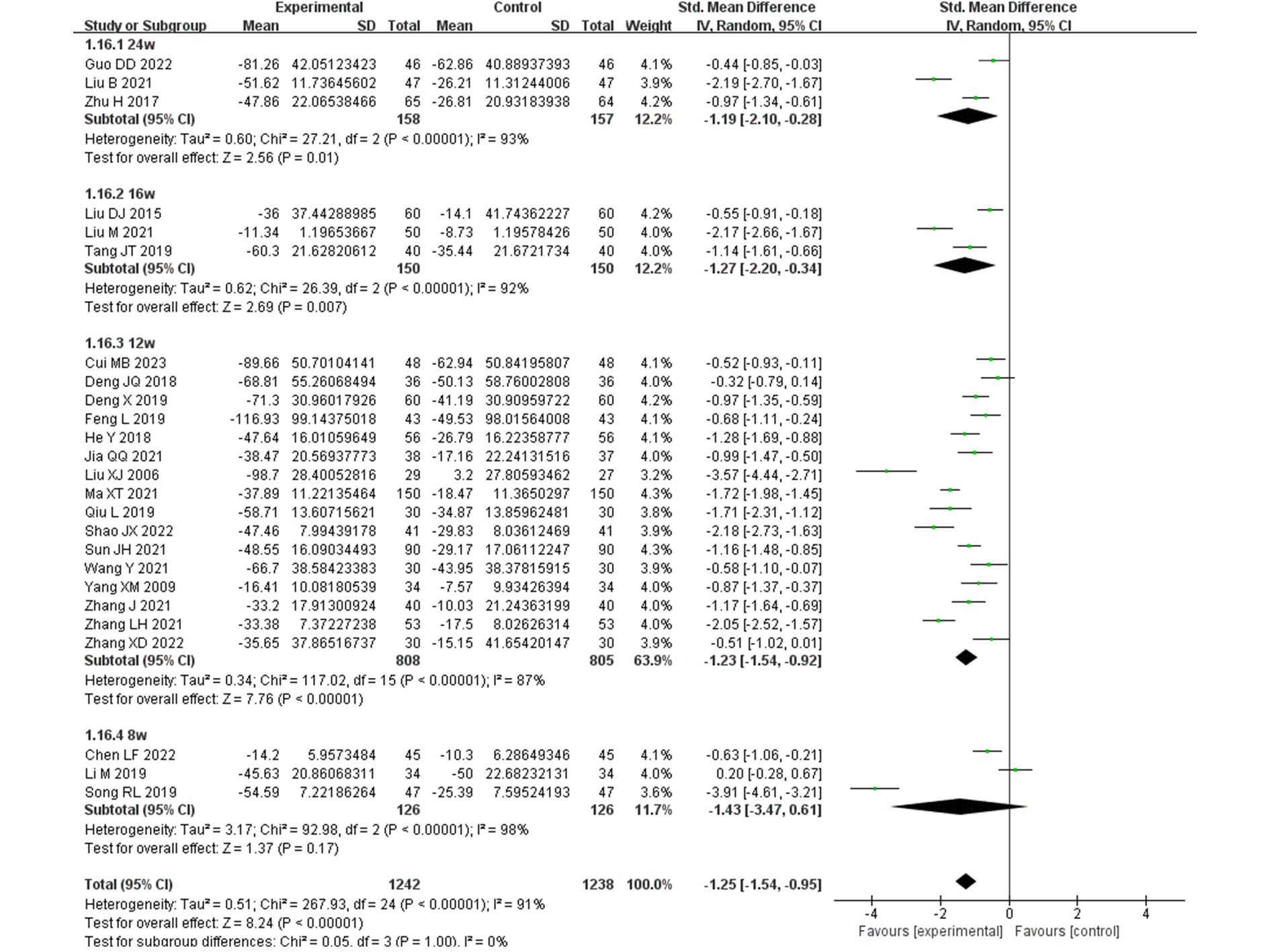

Supplement: Supplementary file 5 [file Image2.TIF]

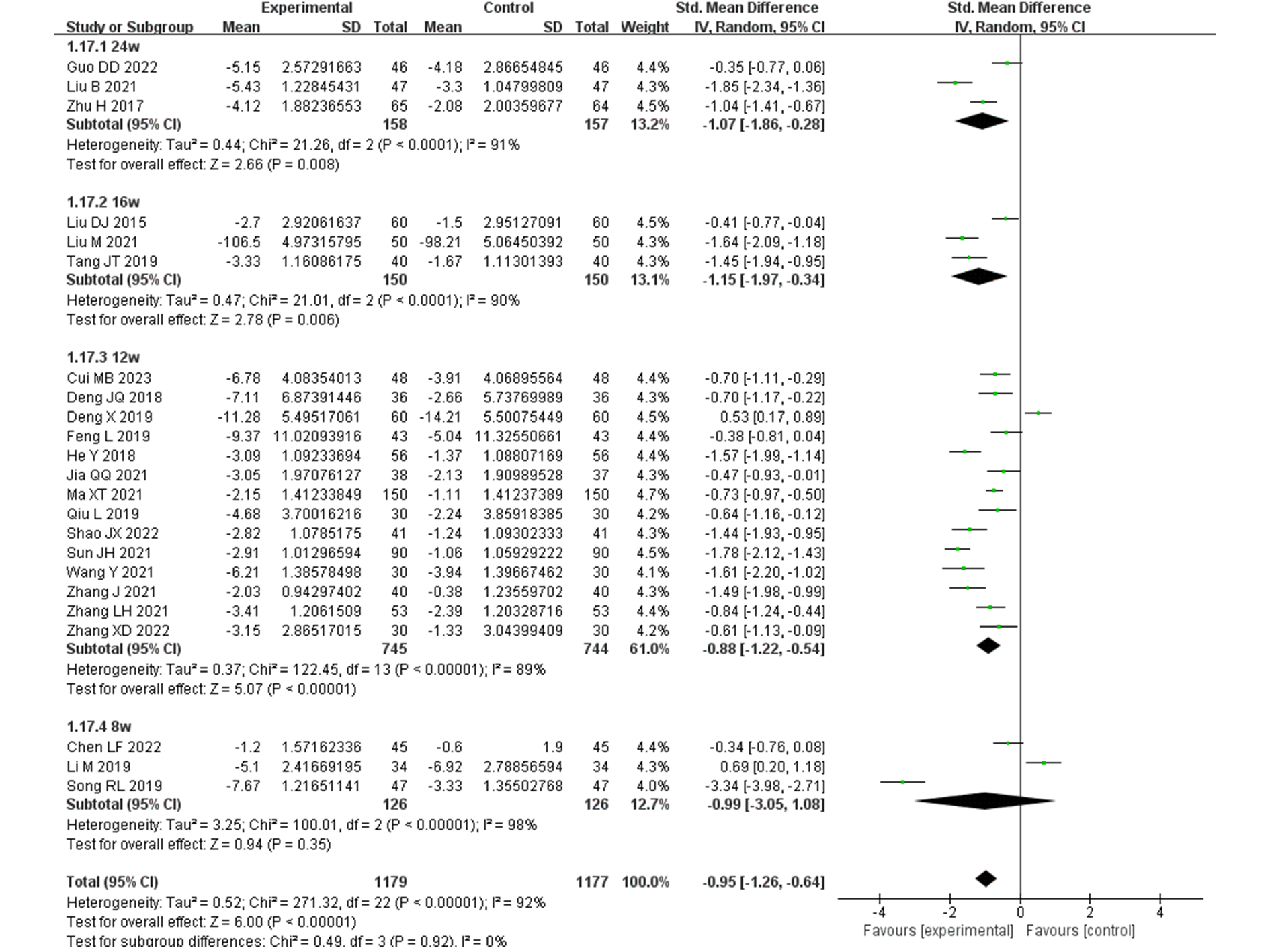

Supplement: Supplementary file 6 [file Image1.TIF]

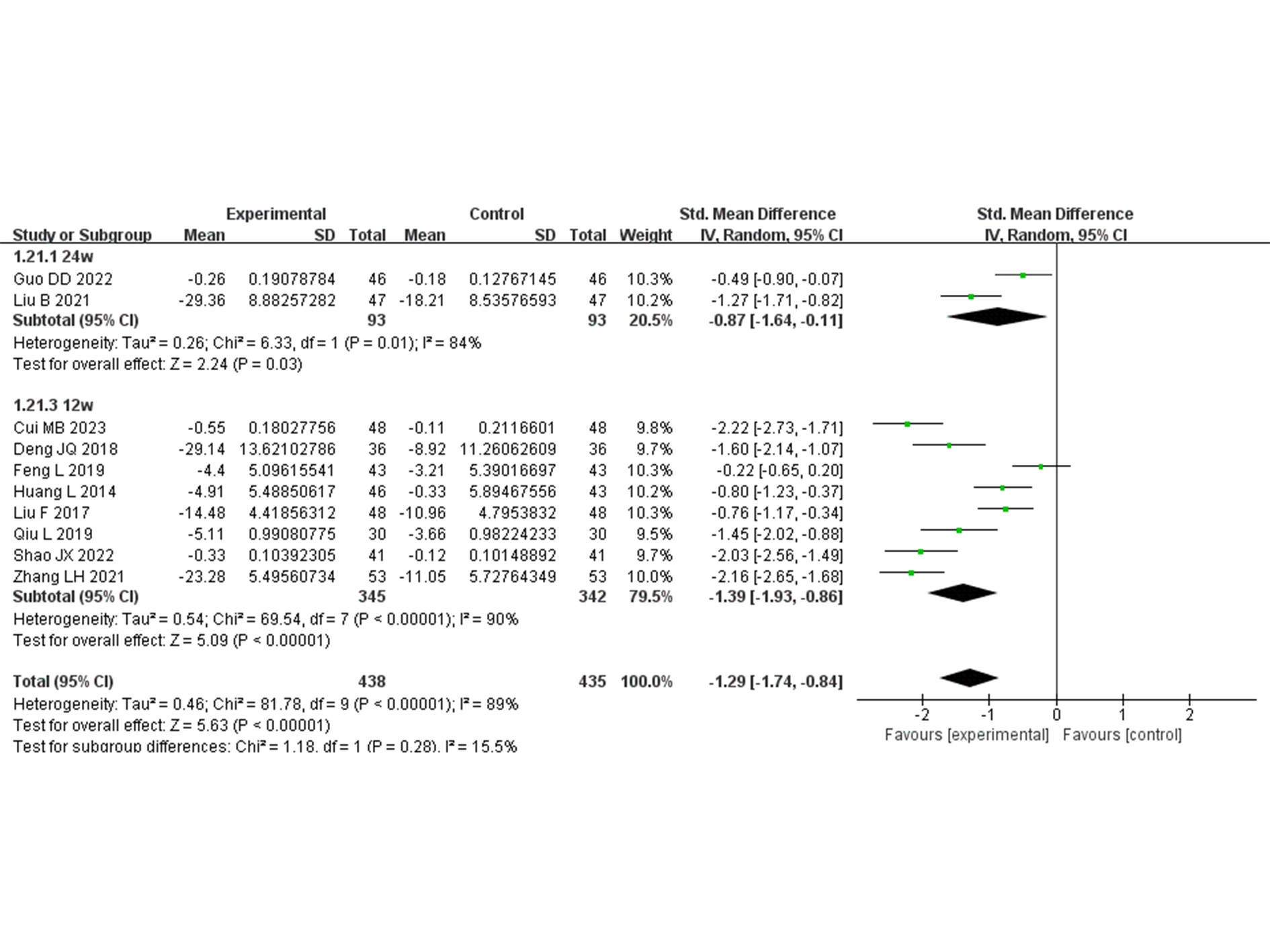

Supplement: Supplementary file 7 [file Image5.TIF]
