# Supplementary material for: Use of bailing capsules (cordyceps sinensis) in the treatment of chronic kidney disease: a meta-analysis and network pharmacology
Source: Front Pharmacol. 2024 Apr 5;15:1342831. doi: 10.3389/fphar.2024.1342831 (PMC11026558; doi:10.3389/fphar.2024.1342831)
Supplement: Supplementary file 2 [file Presentation1.pdf]

Supplement Figure 1. Comparative forest plots of treatment duration subgroups. I^2 and P were used as heterogeneity tests. Forest plot showing the effect of bailing on the outcome of BUN level.

Supplement Figure 2. Comparative forest plots of treatment duration subgroups. I^2 and P were used as heterogeneity tests. Forest plot showing the effect of bailing on the outcome of Scr level.

Supplement Figure 3. Comparative forest plots of treatment duration subgroups. I^2 and P were used as heterogeneity tests. Forest plot showing the effect of bailing on the outcome of 24UP level.

Supplement Figure 4. Comparative forest plots of treatment duration subgroups. I^2 and P were used as heterogeneity tests. Forest plot showing the effect of bailing on the outcome of Hs-CRP level.

Supplement Figure 5. Comparative forest plots of treatment duration subgroups. I^2 and P were used as heterogeneity tests. Forest plot showing the effect of bailing on the outcome of TNF-α level.

Supplement Figure 6. Comparative forest plots of treatment duration subgroups. I^2 and P were used as heterogeneity tests. Forest plot showing the effect of bailing on the outcome of IL-6 level.
